# circFBXO7/miR-96-5p/MTSS1 axis is an important regulator in the Wnt signaling pathway in ovarian cancer

**DOI:** 10.1186/s12943-022-01611-y

**Published:** 2022-06-29

**Authors:** Mengting Wu, Qiongzi Qiu, Qing Zhou, Jia Li, Juze Yang, Chengcai Zheng, Aoran Luo, Xufan Li, Honghe Zhang, Xiaodong Cheng, Weiguo Lu, Pengyuan Liu, Bingjian Lu, Yan Lu

**Affiliations:** 1 Zhejiang Provincial Key Laboratory of Precision Diagnosis and Therapy for Major Gynecological Diseases, Women’s Hospital and Institute of Translational Medicine, Zhejiang University School of Medicine, Hangzhou, 310006 Zhejiang China; 2 Key Laboratory of Precision Medicine in Diagnosis and Monitoring Research of Zhejiang Province, Sir Run Run Shaw Hospital and Institute of Translational Medicine, Zhejiang University School of Medicine, Hangzhou, Zhejiang, 310016 China; 3 Department of Pathology, Research Unit of Intelligence Classification of Tumor Pathology and Precision Therapy, Chinese Academy of Medical Sciences, Zhejiang University School of Medicine, Hangzhou, 310058 Zhejiang China; 4 Cancer Center, Zhejiang University, Hangzhou, 310013 Zhejiang China; 5 Women’s Reproductive Health Key Laboratory of Zhejiang Province, Women’s Hospital, Zhejiang University School of Medicine, Hangzhou, 310006 Zhejiang China

**Keywords:** circFBXO7, Ovarian cancer, miR-96-5p, MTSS1, Wnt/β-catenin signaling

## Abstract

**Background:**

CircRNAs are a novel class of evolutionarily conserved noncoding RNA molecules that form covalently closed continuous loop structures without 5′ caps and 3′ poly(A) tails. Accumulating evidence suggests that circRNAs play important regulatory roles in cancer and are promising biomarkers for cancer diagnosis and prognosis, as well as targets for cancer therapy. In this study, we identify and explore the role of a novel circRNA, circFBXO7, in ovarian cancer.

**Methods:**

rRNA-depleted RNA-sequencing was performed to identify differentially expressed circRNAs between ovarian cancerous and normal tissues. qRT-PCR and single-molecule RNA in-situ hybridization was used to quantify circFBXO7 expression in tumor tissues. The association of circFBXO7 expression with patient prognosis was evaluated by Kaplan–Meier survival analysis. The biological function of circFBXO7 was also investigated using loss-of-function and gain-of-function assays in vivo and in vitro. Luciferase reporter and TOP/FOP-Flash reporter assays were then conducted together with RNA immunoprecipitation and western blot to assess the circFBXO7/miR-96-5p/MTSS1/Wnt/β-catenin axis.

**Results:**

circFBXO7 was downregulated in ovarian cancer which was associated with poor prognosis. Biologically, circFBXO7 overexpression significantly suppressed ovarian cancer cell proliferation, migration, and invasion in vitro, and inhibited tumor growth and metastasis in vivo, whereas its knockdown exerted an opposite role. Mechanistically, circFBXO7 functioned as a competing endogenous RNA for miR-96-5p to regulate the expression of MTSS1. Consequently, downregulation of MTSS1 led to excessive accumulation of β-catenin and increased phosphorylation of GSK3β, leading to the translocation of β-catenin to the nucleus, thereby activating the Wnt/β-catenin signaling pathway and ultimately promoting ovarian cancer progression.

**Conclusions:**

Our findings indicate that circFBXO7 acts as a bone fide tumor suppressor in ovarian cancer and that the circFBXO7/miR-96-5p/MTSS1 axis is an important regulator in the Wnt/β-catenin signaling pathway which may provide a promising target for ovarian cancer therapy.

**Supplementary Information:**

The online version contains supplementary material available at 10.1186/s12943-022-01611-y.

## Background

Circular RNAs (circRNAs) are a newly discovered class of non-coding RNA that are widely expressed and abundant in eukaryotes. In contrast to traditional linear mRNAs, circRNAs lack 5′ caps and 3′ poly (A) tails, where the 5′ and 3′ ends are formed into continuous closed loop structure by cyclic covalent bonds [1, 2]. Due to this specialized structure, circRNAs escape the deadenylation, decapping and degradation normally caused by miRNA regulation, and are therefore highly stable and conservative [3]. With the development of high-throughput sequencing technology twinned with various bioinformatics methods, increasing numbers of circRNA molecules are now being discovered and described. According to their origins, current classification techniques divide circRNAs into three categories, exonic circRNA (ecircRNA), intronic circRNA (ciRNA), and exon-intron circRNA (EIciRNA) [4, 5].

Three main types of circRNA mechanisms have also been identified where they either act as microRNA (miRNA) molecular sponges to regulate gene expression [6], or to bind RNA-binding proteins (RBPs) [7], or are themselves translated into proteins [8]. Many such functions have been linked to cancer regulation. circMAT2B, for example, has been reported to act as a sponge for miR-338-3p to promote glycolysis and hepatocellular carcinoma progression by activating the circMAT2B/miR-338-3p/PKM2 axis under hypoxia [9]. circTP63 competitively binds to miR-873-3p and prevents miR-873-3p from degrading FOXM1, thereby upregulating CENPA and CENPB, and ultimately promoting lung squamous cell carcinoma progression [10]. circHuR interacts with CCHC-type zinc finger nucleic acid binding protein (CNBP) and inhibits CNBP binding to HuR promoter, thereby suppressing gastric cancer progression by inhibiting CNBP transactivation [11]. circZNF609 is formed by the circularization of the second exon of the ZNF609 gene and binds to polyribosomes, suggesting that it has the potential to encode proteins. In one study, by constructing a circZNF609 overexpression vector a 3xFLAG coding sequence was added upstream of the stop codon enabling the transfected cells produced a marker protein. This was used to demonstrate that circZNF609 has a translation function [12]. circSMO, formed from exons 3–6 of the G protein-coupled-like receptor smoothened (SMO) gene, is also reported to encode a novel protein SMO-193aa, that is critical for Hedgehog signaling and drive glioblastoma tumorigenesis [13]. These and many other studies have demonstrated circRNAs as a significant new class of biomarkers which have aspects of clinical importance for disease diagnosis, prognosis and treatment [14, 15]. However, the molecular mechanisms of circRNAs, particularly those relating to carcinogenesis, require further investigation.

Ovarian cancer is one of the most common malignant tumors, ranking fifth for overall female cancer deaths, and with the second highest incidence rate and the highest mortality rate for cancers of the female reproductive system [16]. Early symptoms of ovarian cancer are not obvious and there is a clear lack of reliable early diagnostic biomarkers resulting in most patients being diagnosed at an advanced stage for this cancer. Unlike other types of tumors, ovarian cancer is prone to extensive metastasis in the pelvic and abdominal cavity [17]. Identification of early diagnostic and therapeutic biomarkers critically involved in ovarian cancer is, therefore, of great clinical significance.

In this study, we performed differential expression analysis of circRNAs between ovarian cancerous and normal tissues using rRNA-depleted deep sequencing, and identified a novel circFBXO7 in ovarian cancer, which is produced by back-splicing of exons 2, 3, and 4 of pre-mRNA FBXO7. FBXO7 encodes an adaptor protein in the ubiquitin protein ligase complex called SCF (SKP1-cullin-F-box) that recognizes and mediates phosphorylation-dependent substrate ubiquitination. FBXO7 protein can regulate several key cellular processes by interacting with multiple target proteins, including synapse neuroplasticity [18], cell proliferation regulation [19] and circadian rhythm maintenance [20]. Mutations within FBXO7 have been found to cause Parkinson’s disease and multiple system atrophy [21]. However, FBXO7 exhibited no differential expression between ovarian tumors and normal tissues and was not associated with patient prognosis, whereas circFBXO7 was significantly downregulated in ovarian cancer tissues and positively correlated with better prognosis. Our mechanistic analysis further revealed that circFBXO7 acts as a competing endogenous RNA (ceRNA) of miR-96-5p to regulate the expression of MTSS I-BAR domain containing 1 (MTSS1) and then inhibit the activity of Wnt/β-catenin signaling pathways, thereby suppressing the progression of ovarian cancer.

## Materials and methods

### rRNA-depleted RNA-seq for detecting circRNAs in ovarian cancerous and normal tissues

This study was reviewed and approved by the Ethnics Committees of Women’s Hospital of Zhejiang University School of Medicine (Hangzhou, China). 27 tumor tissues from ovarian cancer patients and 26 normal ovarian tissues from patients with benign gynaecological diseases were collected with permission (Table S1). Total RNA was extracted using the Trizol reagent (Invitrogen, Carlsbad, CA, USA). RNA integrity was assessed using the Agilent 2100 Bioanalyzer System (Agilent Technologies, Palo Alto, CA, USA). Before constructing the complementary DNA library, approximately 1 μg total RNA was treated with a NEBNext rRNA Depletion Kit (NEB, Ipswich, MA, USA, Cat# E6318) to remove ribosomal RNA (rRNA). Subsequently, strand-specific RNA-seq libraries were prepared using the NEBNext Ultra RNA Library Prep Kit (NEB, Cat# E7420) and were sequenced using a HiSeq X10 sequencer (Illumina, San Diego, CA, USA) with paired-end reads of length 2 × 150 bp. Approximately, 129 million reads were generated for each sequencing library (Table S2).

### Identification of circRNAs

The output RNA-Seq sequence reads were pre-processed using Trim Galore (http://www.bioinformatics.babraham.ac.uk/projects/trim_galore/). Adapters and sequences with low quality (base quality < 20) were removed before analysis. The trimmed sequence reads were mapped to the human genome (hg19) and the gene annotation database (Ensembl genes v75, www.ensembl.org) using TopHat2 (v 2.0.13) [22]. All the unmapped reads were then used to identify circRNAs using CIRCexplorer2 [23]. The expression level of circRNA was estimated as the ratio of the number of back-spliced junction reads to the maximum number of reads spanning the linear-spliced junction of the same exon(s) in each library. Any expression differences in circRNAs between tumor and normal samples was examined using Students’ *t*-test in the R statistical package. To identify circRNAs expressed independently of their parental genes, only circRNAs with no significant correlation (*P*-value > 0.05) with their parental genes were selected.

### Cell culture

Four human ovarian cancer cells (A2780, MDAH2774, OV90 and SKOV3) and HEK 293 T cells were cultured at 37 °C in 5% CO_2_. A2780 was cultured in RPMI-1640 media (GIBCO, Carlsbad, CA, USA) supplemented with 10% FBS (GIBCO), penicillin (100 U/ml) and streptomycin (100 ng/ml). MDAH2774 and 293 T were cultured in DMEM media (GIBCO) supplemented with 10% FBS (GIBCO), penicillin (100 U/ml) and streptomycin (100 ng/ml). OV90 was grown in 1:1 mixture of MCDB 105 medium and 199 medium (GIBCO) supplemented with 15% FBS, penicillin (100 U/ml) and streptomycin (100 ng/ml). SKOV3 was cultured in McCoy’s 5A media (BI) supplemented with 10% FBS (GIBCO), penicillin (100 U/ml) and streptomycin (100 ng/ml).

### Cell transfection

The pLO5-circFBXO7 overexpression vector and pLO5-ciR control vector were purchased from GENESEED (Guangzhou, China). Lentivirus particles were generated in 293 T cells which had been co-transfected with the envelope plasmid pCMV-VSVG, the packaging plasmids psPAX2, and pLO5-circFBXO7 overexpression or pLO5-ciR control plasmids, using lipofectamine 3000 (Invitrogen, Carlsbad, CA, USA). Cells were infected with filtered lentivirus plus 8 μg/mL polybrene (Sigma-Aldrich, St. Louis, MO, USA) for 48 hours and then treated with 5 μg/mL puromycin (InvivoGen, San Diego, CA, USA) for more than 7 days to obtain stably transfected cells. The whole CDS sequence of MTSS1 was then cloned into a pcDNA3.1 vector to construct an MTSS1 overexpression vector. Small interference RNAs (siRNAs) that target the junction sequence of circFBXO7 and MTSS1 were designed and synthesized by GenePharma (Shanghai, China). Cells were transfected with these siRNAs using GeneMute™ reagent (SignaGen Laboratories, Rockville, MD, USA). Information on siRNAs used the study is shown in Table S3.

### RNA-seq for circFBXO7 overexpression and control cells

Total RNAs of three repeated experiments were extracted from A2780 and MDAH2774 ovarian cancer cells transfected with circFBXO7 overexpression plasmid or control plasmid. mRNA sequencing libraries of these transfected cells (Table S4) were prepared using a TruSeq RNA Sample Preparation Kit from Illumina as we described previously [24]. Sequencing data were preprocessed and mapped (as before) to the human genome (hg19) using TopHat2 (v 2.0.13) [22]. Transcripts were then constructed and identified using Stringtie2 (v2.1.0) [25]. Differentially expressed genes (DEGs) (adjusted *P*-values < 0.05) between circFBXO7 overexpression and mock cells were detected using Cuffdiff2 (v2.2.1) [26].

### CCK-8 assay and colony formation

For CCK-8 cell viability assays, an equal number of cells were plated into 96-well plates using 5 wells for replicates. These were then incubated at 37 °C/5% CO2 for 0 h, 24 h, 48 h, 72 h, 96 h or 120 h, respectively. Absorbance was measured at a wavelength of 450 nm after adding CCK-8 (Dojindo Laboratories, Kumamoto, Japan) for 2 h using a microplate reader (Bio-Rad, Model 680, Hercules, CA, USA). For colony formation assays, ovarian cancer cells were plated into 12-well plates with 2000 cells/well. The cells were cultured for 1 week, and then fixed with methanol and stained with crystal violet.

### Cell migration assay

The ovarian cancer cells (1 × 10^5^ cells for A2780, and 3 × 10^4^ cells for MDAH2774/SKOV3/OV90) were transfected with siRNA or overexpression plasmids for 24 h, and then cultured with serum-free medium for 24 h. The starved cells were plated with 300 μL cell suspension into the upper transwells and placed in the above 500 uL media containing 10% FBS in 24-well plates. Plates were incubated at 5% CO2 and 37 °C for 24 h. Cells that had migrated into the bottom chamber were then fixed with methanol and stained with 0.1% crystal violet. Images were captured from each membrane and the number of migratory cells was counted under a microscope.

### Real-time quantitative RT-PCR

Total RNA was extracted using Trizol (Invitrogen) and 1 μg total RNA was reverse transcribed into cDNA in a reaction volume of 20 μL using SuperScript II (Vazyme, Nanjing, China). The amplification reaction volume was 10 μL containing SYBR Green PCR Master Mix (Vazyme), 1 μL cDNA, and amplification primers. Information of primers is shown in Table S3.

### Western blot

Cells were suspended in RIPA with PMSF, and the cellular lysates were then centrifuged at 14,000 rpm for 30 min. The protein concentration of the supernatant was determined using the BCA assay (Thermo Fisher Scientific, Waltham, MA, USA). Equal amount of proteins (40 μg) was added to 10% sodium dodecyl sulfate-polyacrylamide gel electrophoresis (SDS-PAGE) gel. The proteins were then transferred onto a polyvinylidene fluoride (PVDF) membrane (300 mA, 2 h). The membrane was blocked with 5% skim milk and incubated with the primary antibodies overnight. Membranes treated with primary antibodies were then washed three times with TBST and incubated with an anti-rabbit or anti-mouse HRP-linked antibody at a 1:2000 dilutions for 1 h at room temperature. After washing three times with TBST the proteins were detected by western blot using ECL. The signal was visualized using ECL reagent and photographed using the ChemiDoc Touch Imaging System (Bio-Rad, Hercules, CA, USA). Information of antibodies for VIM, snail, β-catenin, p-GSK3β, MTSS1, β-actin and GAPDH is shown in Table S5.

### Single-molecule RNA in-situ hybridization

The expression of circFBXO7 in ovarian cancer tissues was evaluated using a BaseScope Assay (Advanced Cell Diagnostics (ACD), Newark, CA, USA). A 1ZZ BaseScope probe targeting the junction sequences of circFBXO7 was then designed. Formalin-fixed, paraffin-embedded (FFPE) tissue samples from ovarian cancer patients from Women’s Hospital of Zhejiang University School of Medicine were prepared (Table S1). BaseScope assays were performed using a BaseScope Reagent Kit V2-RED (ACD, Cat# 323900) following the manufacturer’s protocol. Chromogenic detection was performed using BaseScope Fast RED followed by counterstaining with hematoxylin (American MasterTech Scientific, Lodi, CA). At 20× magnification, the number of visible red dots in 10 randomly scanned regions of each image was used to measure the expression of circFBXO7 in each FFPE tissue.

### Survival analysis

The expression of FBXO7 (in format of FPKM) from ovarian cancer RNA-seq data in The Cancer Genome Atlas (TCGA) was downloaded using the GDC data portal (https://portal.gdc.cancer.gov/) [27]. Prior to survival analysis, the FBXO7 expression was normalized using log2 transformed FPKM value. The expression of circFBXO7 in FFPE tissues from patients with ovarian cancer was scored by Basescope assay (Table S1).

The event of overall survival was defined as death, while the relapse-free survival was ended by any disease recurrence or death. The median overall survival time of patients in TCGA is 46 months, ranging from 1 to 183 months, and the median relapse-free survival time is 18 months, ranging from 1 to 183 months. The median overall survival time of patients in our cohort is 83 months, ranging from 6 to 177 months, and the median relapse-free survival time is 38 months, ranging from 1 to 177 months. Patient samples were collected from 1992 to 2009 in the TCGA cohort, of which 88.14% were Caucasian. While in our cohort, samples were collected from 2006 to 2012, and all patients were Asian. The median age at diagnosis for the TCGA and our cohort was 59 and 53 years, respectively. 90.23% patients were diagnosed with advanced stages (Stage III or IV) in the TCGA cohort, compared with 61.43% in our cohort. As for the treatment represented by adjuvant paclitaxel therapy, there was no significant difference between two cohorts (*P* = 0.78, Chi-squared test).

Patients with ovarian cancer were divided into low- and high-risk groups according to the median expression levels of FBX7 or circFBXO7. Survival distributions in different groups were visualized using Kaplan-Meier curves, and the significance was assessed by a log-rank test. Survival analysis was performed using the R package “survival.”

### Animal experiments

Five-week-old female athymic nude mice (BALB/c Nude) and NOD/SCID mice were used (5 mice per group). For the local model, MDAH2774 cells with stable circFBXO7 overexpression and negative control (8 × 10^6^ cells) were suspended in 0.1 ml PBS and subcutaneously inoculated into the flanks of the nude mice. Tumor growth was monitored every 2 days and tumor volumes were measured using a caliper and calculated using the following formula: Volume (cm^3^) = (length × width^2^)/2. Four weeks after inoculation, the mice were euthanized adhering to the policy on the humane treatment of tumor-bearing animals. Tumor weights were then measured. For the systemic model, MDAH2774 cells with stable circFBXO7 overexpression and negative control (2 × 10^6^ cells) suspended in 0.1 ml PBS were intraperitoneal injected to NOD/SCID mice. 1.5 mg luciferin (Gold Biotech, St Louis, MO, USA) was administered once a week for 5 weeks, to monitor tumor cell metastases using an IVIS@ Lumina II system (Caliper Life Sciences, Hopkinton, MA, USA). Tumor data were presented as the mean ± standard deviation of five mice at each time point. Differences in tumor weight, volume, and metastasis between circFBXO7 overexpression and negative control groups were examined using a two-tailed Student’s t test. Differences were considered statistically significant at *P* < 0.05. All experiments were performed in accordance with the Guide for the Care and Use of Laboratory Animals (NIH publication 80–23, revised 1996) of Zhejiang University, Hangzhou, China.

### Luciferase report assay

The sequence of circFBXO7 (or the 3′ UTR of MTSS1) was subcloned into the psiCHECK2 vector (Promega, Madison, WI, USA, Cat# C8021). In the mutant vectors, the miR-96-5p binding sites in circFBXO7 or the 3’UTR of MTSS1 were mutated on psiCHECK2 vectors. For the TOP/FOP flash assay, the ovarian cancer cells were transfected with 0.5 μg TOP/FOP plasmid and 12.5 ng Renilla per well using Lipofectamine 3000 in 24-well plates. At the same time, ovarian cancer cells were transfected with circFBXO7 overexpression plasmids or MTSS1 siRNA. The ovarian cancer cells were then transfected with 0.5 μg reporter vector and miR-96-5p mimic per well using Lipofectamine 3000 in 24-well plates. After 24 hours transfection, the cells were lysed with passive lysis buffer (Promega, Cat# E1910), and reporter gene expression was assessed using a Dual Luciferase reporter assay system (Promega, Cat# E1910).

### Cytoplasmic & nuclear fractionation

The nuclear and cytoplasmic fraction of cells was isolated using the PARIS™ kit (Ambion, Austin, TX, USA, Cat#AM1921). Approximately 10^7^ cells were washed with cold PBS, and then resuspended in 600 μL cell fractionation buffer for 5 min on ice. The lysates were then centrifuged at 500×g for 5 min at 4 °C. The resulting supernatant was collected as the cytoplasmic fraction. Precipitation was added with 600 μL cell fractionation buffer for 3 min on ice, the lysates were centrifuged at 500×g for 3 min at 4 °C. After removing the supernatant, 500 μL cell disruption buffer was added to resuspend the pellet to obtain the nuclear fraction.

### RNA immunoprecipitation

An RNA immunoprecipitation (RIP) assay was performed using the Magna RIP™ RNA-Binding Protein Immunoprecipitation Kit (EMD Millipore, Darmstadt, Germany, Cat# 17–700). 2 × 10^7^ cells were lysed in 100 μL RIP lysis buffer with the addition of 0.5 μL of protease inhibitor cocktail and 0.25 μL RNase inhibitor, and then lysed on ice for 5 min. Meanwhile, IgG and AGO2 antibody with protein A/G magnetic beads were incubated at room temperature for an hour. The mixture was incubated with lysates at 4 °C overnight. The beads were washed six times with wash buffer, and then resuspended in protein K buffer for 30 min at 55 °C. After extracting RNA, the abundance of circFBXO7 was measured using qPCR.

### Statistical analysis

All experiments were repeated at least 3 times. Representative experiments are shown. Protein abundances from western blots were quantified using ImageJ (https://imagej.nih.gov/ij/). Data are presented as mean ± standard deviation of three independent experiments. For comparison of two groups, a two-tailed Student’s t test was used. Comparison of multiple groups was made using a one- or two-way ANOVA. Differences were considered statistically significant at *P* < 0.05.

## Results

### CircFBXO7 is downregulated in ovarian cancer and associated with poor prognosis

**Fig. 1:**
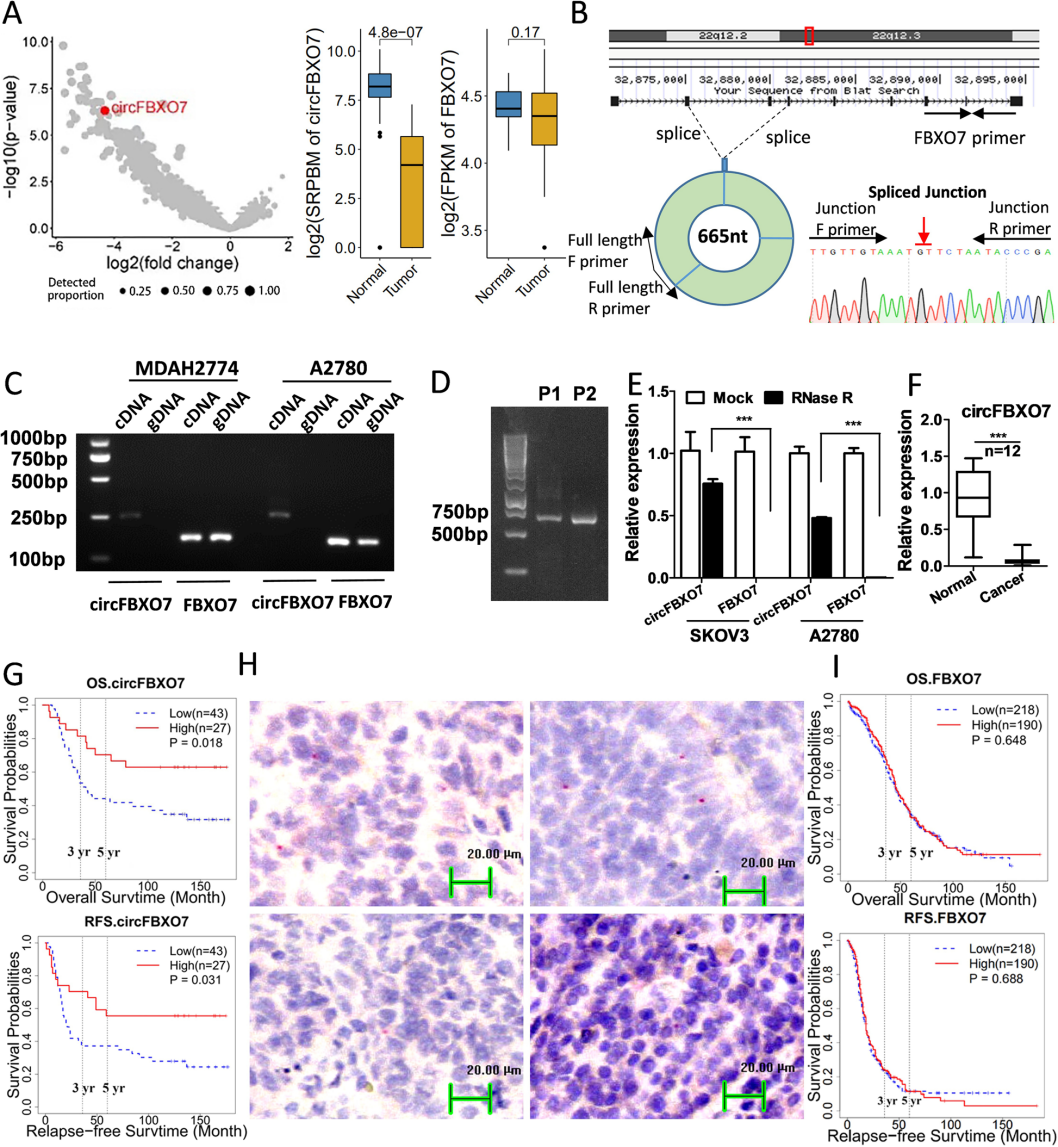
circFBXO7 is downregulated in ovarian cancer and is associated with poor prognosis. **A** Volcano plot showing the relative expression of circRNA in ovarian cancer and normal ovarian tissue. circFBXO7 is marked as a red circle. Expression of circFBXO7 and its parental gene FBXO7 in tumor and normal tissue is shown in the right panel. **B** Schematic diagram of the formation of circFBXO7. circFBXO7 was back-spliced from 3 exons of FBXO7 and its full-length sequence was confirmed by Sanger sequencing. **C** Agarose gel electrophoresis showed circFBXO7 as amplified by divergent primers in cDNA but not in gDNA of A2780 and MDAH2774. **D** The full length of circFBXO7 obtained by two different primers. **E** Relative abundance of circFBXO7 and linear-FBXO7 in TOV21G and A2780 cells treated with RNase R, as determined by qRT-PCR. **F** qRT-PCR analysis of the expression of circFBXO7 in an independent cohort including 12 tumor tissues from ovarian cancer patients and 12 normal tissues from patients with benign gynaecological diseases. **G** Kaplan-Meier survival curve of circFBXO7 expression level in 70 FFPE tissues from ovarian cancer patients. The expression level of circFBXO7 was evaluated using the BaseScope assay. **H** BaseScope images (20× magnification) showing the signal points which measure the expression of circFBXO7. The two upper panels represent the better the prognosis with higher the expressions of circFBXO7, and the two lower panels represent the worse the prognosis with lower expressions of circFBXO7. **I** Kaplan-Meier survival curve of linear FBXO7 expression level of ovarian cancer patients from TCGA. *** *P* < 0.001, ** *P* < 0.01, * *P* < 0.05

To identify the role of circRNAs in ovarian cancer, we analyzed the rRNA-depleted RNA-seq data from 27 tumor tissues from ovarian cancer patients and 26 normal ovarian tissues from patients with gynaecological diseases. Volcano plots (Fig. 1A) display the log2-fold change in circRNA expression between ovarian tumor and normal tissues versus its associated -log10 of the *P*-values. CircFBXO7 was identified as one of the most significantly downregulated circRNAs in ovarian cancer, while its parental gene FBXO7 lacked any significant differential expression between ovarian tumors and normal ovarian tissues. circFBXO7 is spliced from exons 2, 3 and 4 of FBXO7 on the sense strand of chromosome 22 (from 32,874,967 to 328,81,196 bp, hg19 genome build). To verify the existence of circFBXO7, divergent primers were designed to amplify circFBXO7 in different ovarian cancer cells (Fig. 1B). Agarose gel electrophoresis showed that whilst circFBXO7 was amplified by divergent primers only in the cDNA of A2780 and MDAH2774, FBXO7 was detected in both cDNA and genomic DNA (Fig. 1C). Two pairs of different primers, in which the first base of the forward and reverse primers occur next to each other, were then designed to confirm the full length of circFBXO7 (Fig. 1D). CircFBXO7 contains 665 nucleotides and its full-length sequences are given in Fig. S1. As expected, circFBXO7 displayed increased tolerance against RNase R exonuclease digestion, as compared to FBXO7, suggesting that circFBXO7 bears a circular structure lacking a 5′ cap and 3′ tail (Fig. 1E). Furthermore, qRT-PCR results from 12 independent ovarian cancer tissue samples confirmed the downregulation of circFBXO7 in tumor tissues compared to 12 corresponding normal ovarian tissue samples (Fig. 1F). Basescope assays using an 1ZZ BaseScope probe were used to assess circFBXO7 expression in FFPE tissues from ovarian cancer patients with known clinical outcomes. Kaplan–Meier survival analysis indicated that the lower expression of circFBXO7 was associated with shorter overall survival and relapse–free survival in ovarian cancer patients (Fig. 1G and H). However, the expression of FBXO7 was not significantly corelated with patient prognosis (Fig. 1I). Taken together, these experiments validated the existence of circFBXO7 detected by rRNA-depleted RNA-seq in ovarian tumor tissues and demonstrated the clinical significance of circFBXO7 as a potential diagnostic and prognostic biomarker for ovarian cancer patients.

### CircFBXO7 acts as a tumor suppressor in ovarian cancer

**Fig. 2:**
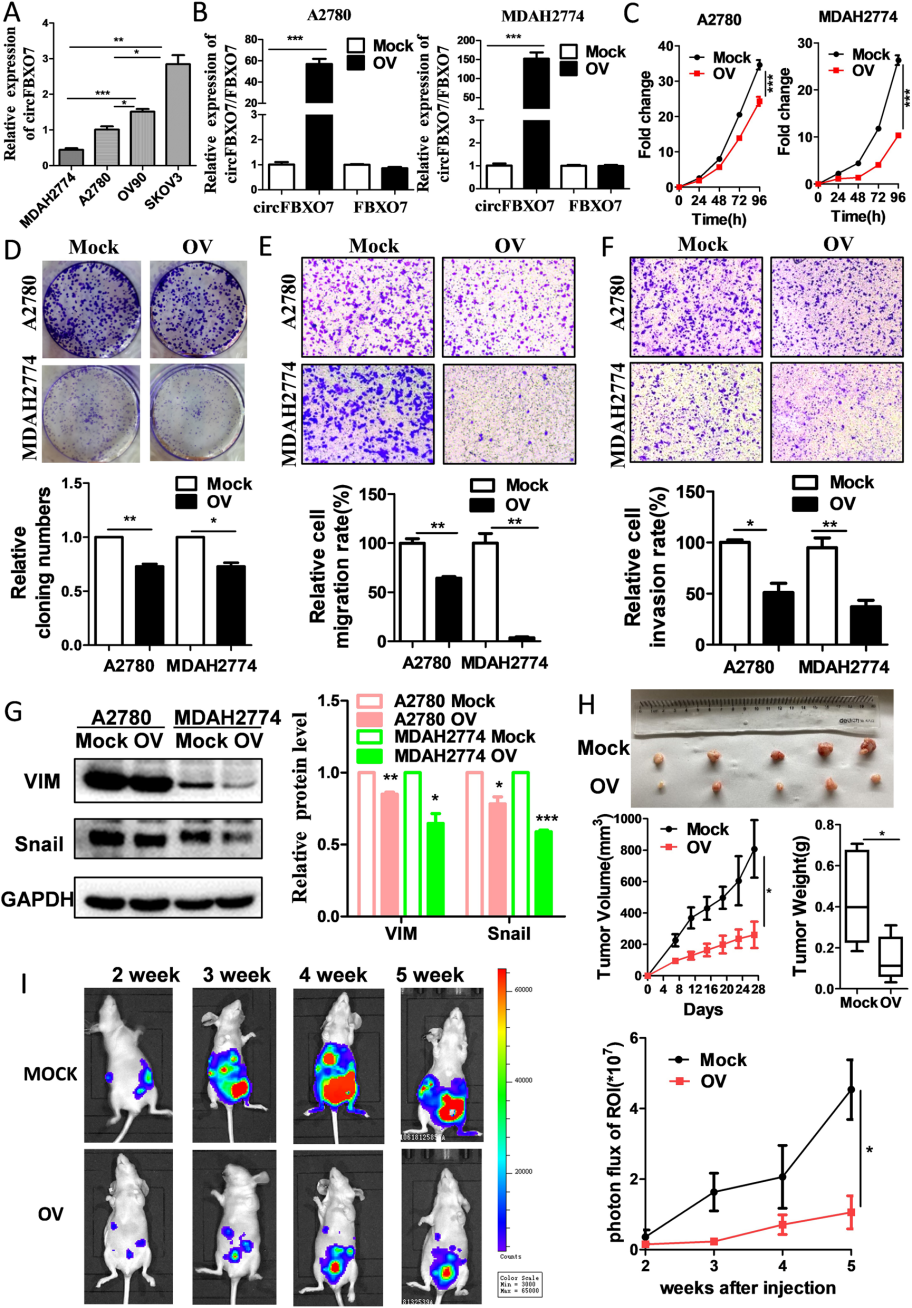
circFBXO7 acts as a tumor suppressor and inhibits the proliferation, migration and invasion of ovarian cancer cells. **A** circFBXO7 expression in different ovarian cancer cell lines, assessed by qRT-PCR. **B** The expression of circFBXO7 and linear-FBXO7 in A2780 and MDAH2774 cells transfected with circFBXO7 or mock plasmids, as assessed by qRT-PCR. **C** Growth curve of A2780 and MDAH2774 cells transfected with circFBXO7 or mock plasmids as measured using CCK-8 assays. **D** Proliferation of A2780 and MDAH2774 cells transfected with circFBXO7 or mock plasmids as determined by colony formation assay. **E** Migration ability of A2780 and MDAH2774 cells transfected with circFBXO7 or mock plasmids. **F** Invasion ability of A2780 and MDAH2774 cells transfected with circFBXO7 or mock plasmids. **G** Western blot analysis of the expression of VIM and snail in A2780 and MDAH2774 cells transfected with circFBXO7 or mock plasmids. **H** Tumor volume and weight of nude mice injected with circFBXO7 and mock plasmids in right and left side, respectively. **I** Intraperitoneal injection of circFBXO7 overexpression cells into nude mice. *** *P* < 0.001, ** *P* < 0.01, * *P* < 0.05

As described above, circFBXO7 expression is significantly downregulated in cancer tissues and its downregulation is associated with a poor prognosis in ovarian cancer, suggesting that circFBXO7 may act as a tumor suppressor in ovarian cancer. To test this hypothesis, we evaluated its potential tumor suppressing role in vitro and in vivo. Firstly, we examined the expression level of circFBXO7 in several ovarian cancer cells (Fig. 2A). We chose A2780 and MDAH2774, with lower expression of circFBXO7, to transfect lentiviral overexpression vectors, while SKOV3 and OV90, with higher expression of circFBXO7, were transfected with siRNAs targeting spliced junctions. circFBXO7 was significantly upregulated after transfection of the overexpression vector in A2780 and MDAH2274 cells, whereas the FBXO7 mRNA levels showed no obvious changes (Fig. 2B). Conversely, circFBXO7 was significantly reduced after transfecting the siRNAs in SKOV3 and OV90 cells, whereas the FBXO7 mRNA levels showed no significant changes (Fig. S2A). Consequently, the upregulation of circFBXO7 significantly inhibited proliferation (Fig. 2C and D), migration (Fig. 2E) and invasion (Fig. 2F), whereas the depletion of circFBXO7 significantly promoted all three of these aspects, in ovarian cancer cells (Fig. S2B-D). Overexpression of circFBXO7 decreased the expression of SNAIL1 and vimentin (Fig. 2G), indicating that epithelial-mesenchymal transitions (EMT) is repressed upon the upregulation of circFBXO7. To further confirm the function of circFBXO7 in vivo, we subcutaneously injected MDAH2774 cells with stable overexpression of circFBXO7, or mock controls, into the nude mice. We observed that the tumor volumes and weights of the circFBXO7 overexpression group were significantly smaller than those of the control groups, suggesting that circFBXO7 can suppress the growth of ovarian cancer cells in vivo (Fig. 2H). Additionally, we conducted intraperitoneal injection of cells with stable circFBXO7 overexpression or mock controls into NOD/SCID mice and monitored the photon flux of metastases by injecting luciferin. It was observed that overexpression of circFBXO7 could inhibit the metastasis of ovarian cancer cells in vivo (Fig. 2I). Taken together, these results suggest that circFBXO7 plays a tumor-suppressive role in ovarian cancer in vitro and in vivo.

### circFBXO7 sponges miR-96-5p and suppresses miR-96-5p activity

**Fig. 3:**
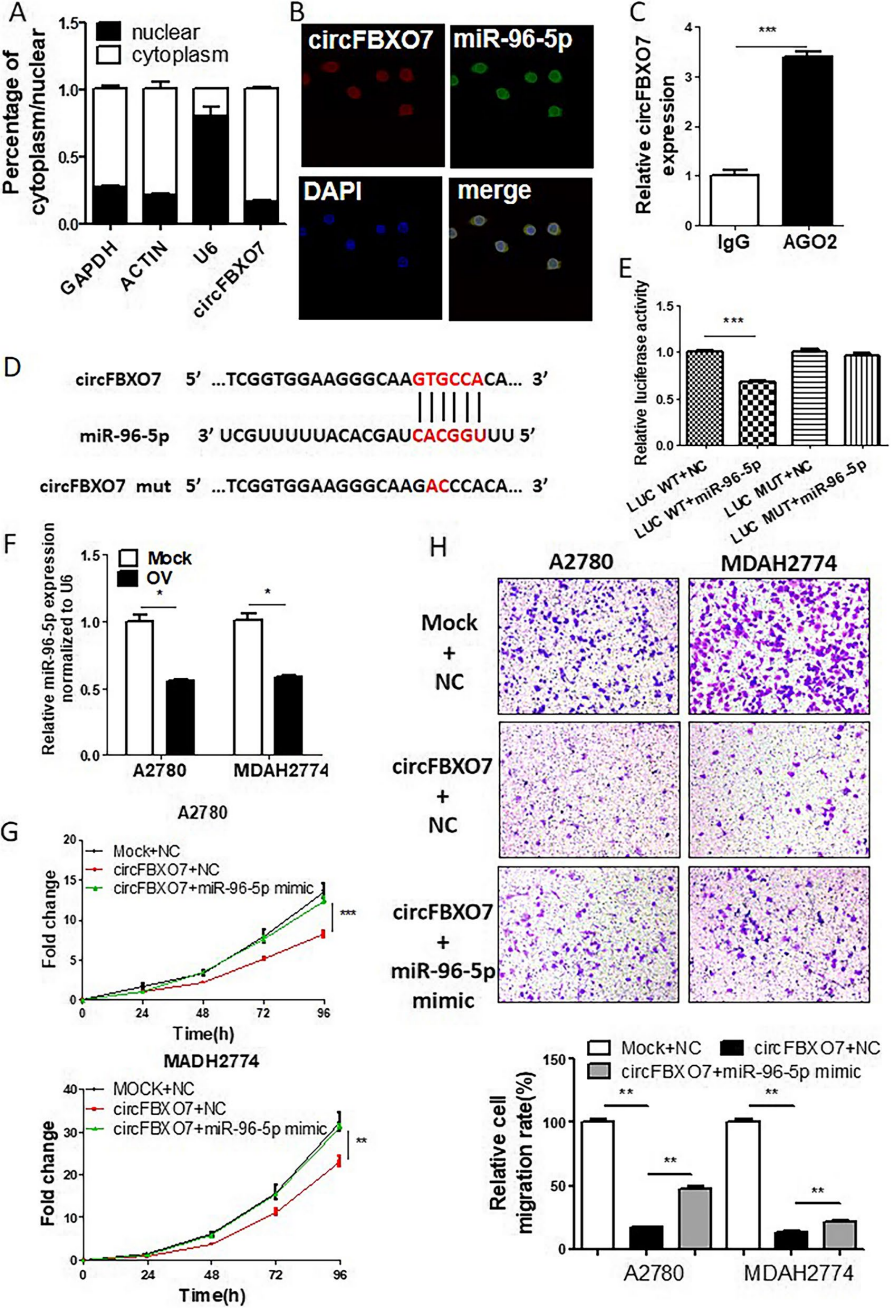
circFBXO7 targets miR-96-5p. **A** circFBXO7 expression in nuclear and cytosolic fractions of ovarian cancer cells assessed by qRT-PCR. U6 was served as a positive control for the nuclear fractions; GAPDH and β-actin as positive control for the cytoplasmic fraction. **B** FISH images of the subcellular localization and expression of miR-96-5p (green) and circFBXO7(red). Nuclei were counterstained with DAPI (blue). **C** RIP experiments were performed in ovarian cancer cells and the coprecipitated RNA was used to quantify circFBXO7 expression using qRT-PCR. **D** Luciferase report vector of wild-type and mutant circFBXO7. **E** Luciferase activity of circFBXO7 wild-type and mutant transfected with miR-96-5p mimics or negative control mimics. **F** miR-96-5p expression by qRT-PCR in A2780 and MDAH2774 cells transfected with circFBXO7 or mock plasmids. **G** Growth curve performed for circFBXO7 overexpression A2780 and MDAH2774 cells transfected with miR-96-5p mimics or negative control mimics. **H** Migration assays performed for circFBXO7 overexpression A2780 and MDAH2774 cells transfected with miR-96-5p mimics or negative control mimics. Migration rates were summarized by histogram in the lower panel. *** *P* < 0.001, ** *P* < 0.01, * *P* < 0.05

Previous studies have shown that known circRNAs can affect the biogenesis of cancers in diverse ways. These include circRNAs functioning as miRNA sponges, binding with RBPs, working as transcription factors, or translating into proteins. To gain a deeper understanding of molecular mechanisms of circFBXO7 in ovarian carcinogenesis, we firstly checked the cellular localization of circFBXO7. Both cytoplasmic & nuclear fractionation and FISH assays revealed that circFBXO7 is predominantly localized in the cytoplasm of ovarian cancer cells (Fig. 3A and B). In addition, an anti-Ago2 RIP assay showed that endogenous circFBXO7 was enriched in the IP fraction compared with IgG, indicating that circFBXO7 can bind to AGO2 protein (Fig. 3C). The results of these assays collectively suggest that circFBXO7 may play a role as a molecular sponge of miRNAs in ovarian cells. Therefore, we investigated the potential of miRNA binding to circFBXO7. Using bioinformatics analysis, we found that circFBXO7 possessed a complementary sequence to the miR-96-5p seed region (Fig. 3D). Luciferase reporter assays showed that only miR-96-5p mimics could remarkably suppress pSICHECK2-circFBXO7 luciferase activity, whereas the luciferase activity was not significantly changed when the binding site of miR-96-5p was mutated (Fig. 3E). The co-localization results from the FISH assay also confirmed a physical interaction between circFBXO7 and miR-96-5p (Fig. 3B). Several previous studies have shown that miR-96-5p promotes cell proliferation and migration in ovarian cancer [28], non-small cell lung carcinomas [29] and head and neck squamous cell carcinomas [30]. Circulating miR-96-5p was also noted as highly expressed in the plasma of breast cancer patients and this could be used as an effective diagnostic biomarker [31]. In our study, upregulation of circFBXO7 decreased the expression level of miR-96-5p in A2780 and MDAH2774 cells (Fig. 3F). To investigate whether the binding of miR-96-5p and circFBXO7 regulates the function of cancer cells, we co-transfected A2780 and MDAH2774 cells with circFBXO7 overexpression vectors and miR-96-5p mimics. Compared to the controls, transfection of miR-96-5p mimics significantly rescued the decreased cell proliferation and migration caused by circFBXO7 overexpression (Fig. 3G and H). Collectively, these results strongly support the suggestion that circFBXO7 binds directly to miR-96-5p and suppresses the activity of miR-96-5p in ovarian cancer cells.

### MTSS1 is a target of miR-96-5p and is indirectly regulated by circFBXO7

**Fig. 4:**
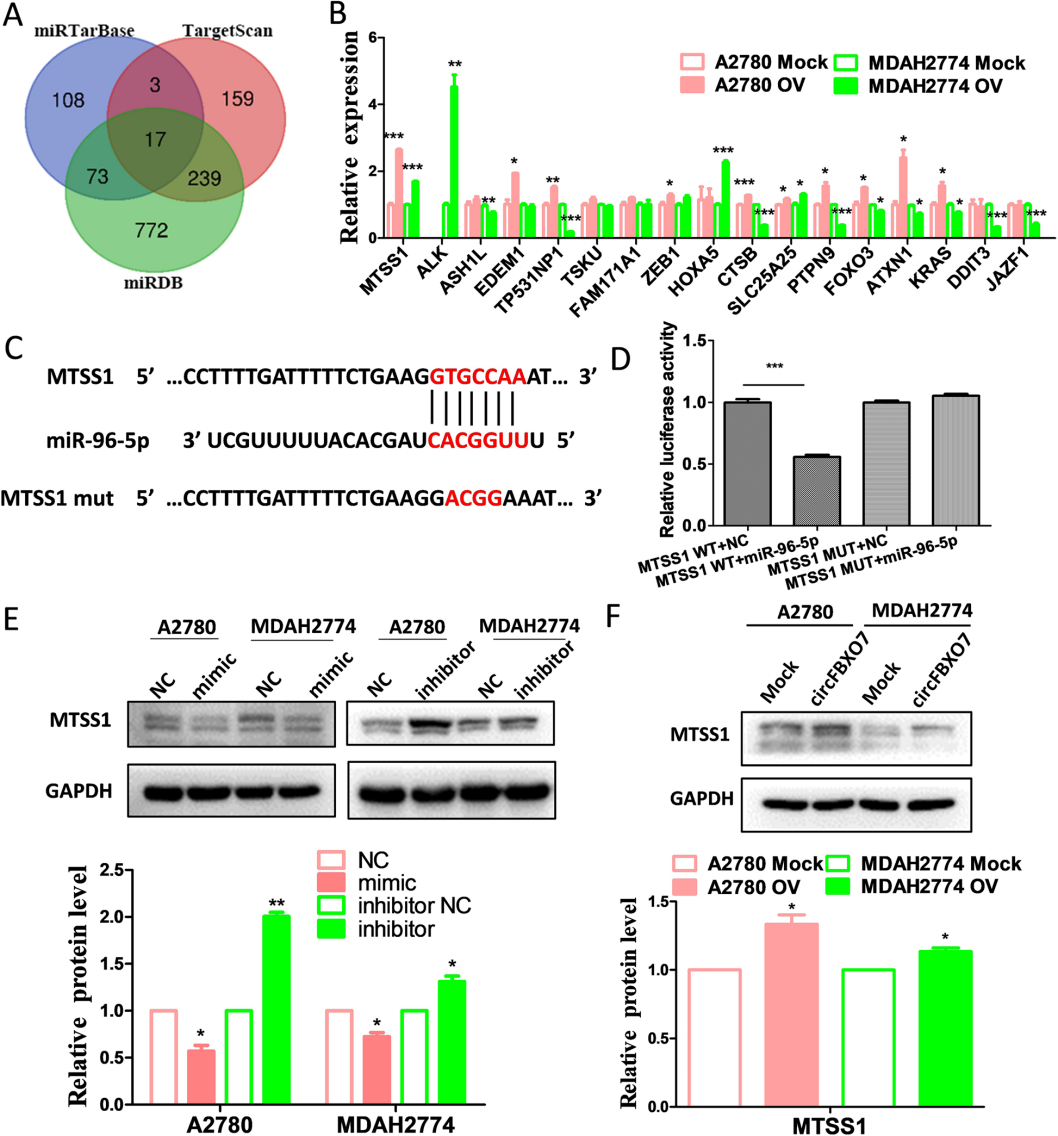
MTSS1 is a target of miR-96-5p. **A** Venn diagram of miR-96-5p targets predicted by miRDB, TarBase and TargetScan. **B** qRT-PCR analysis of potential miR-96-5p target genes that were predicted by all three miRNA target databases (miRTarBase, TargetScan and miRDB) in A2780 and MDAH2774 cells transfected with circFBXO7 and mock plasmids. **C** Luciferase report vector of wild-type and mutant MTSS1. **D** Luciferase activity of MTSS1 wild-type and mutant cells transfected with miR-96-5p mimics and negative control mimics. **E** Western blot analysis of MTSS1 expression in A2780 and MDAH2774 cells transfected with miR-96-5p mimics or negative control mimics, and miR-96-5p inhibitors or negative control inhibitors. **F** Western blot analysis of the expression of MTSS1 in A2780 and MDAH2774 cells transfected with circFBXO7 or mock plasmids. *** *P* < 0.001, ** *P* < 0.01, * *P* < 0.05

To investigate novel downstream molecules, three bioinformatics databases (miRTarBase, TargetScan and miRDB) were used to predict the potential targets of miR-96-5p [32–34]. Seventeen genes were consistently predicted as potential targets of miR-96-5p by all these three databases (Fig. 4A). We evaluated the expression alteration of these genes in A2780 and MDAH2774 cells transfected with circFBXO7 or mock plasmids by qRT-PCR. We observed that MTSS1 had the most consistent significant expression changes upon circFBXO7 overexpression for both cell lines (Fig. 4B). The 3’UTR sequence of MTSS1 was blasted in the miRbase database (v20, http://www.mirbase.org/) to find the binding sites with miR-96-5p. Interestingly, the identical sequence on the miR-96-5p seed region binds to either MTSS1 or circFBXO7 with a seven or six base-pairing, respectively (Fig. 4C and D). Luciferase reporter assays demonstrated that only miR-96-5p mimics could significantly decrease luciferase activity, whereas the luciferase activity was not significantly changed when the binding site was mutated (Fig. 4D). Moreover, transfecting the miR-96-5p mimic significantly decreased the MTSS1 protein level, whereas transfecting the miR-96-5p inhibitor led to its increase (Fig. 4E). In addition, overexpressing circFBXO7 in A2780 and MDAH2774 cells significantly increased both MTSS1 mRNA and protein levels (Fig. 4B and F). Taken together, these findings demonstrate that circFBXO7 can act as a ceRNA for miR-96-5p to regulate MTSS1 expression.

### MTSS1 suppresses the proliferation and migration of ovarian cancer cells

**Fig. 5:**
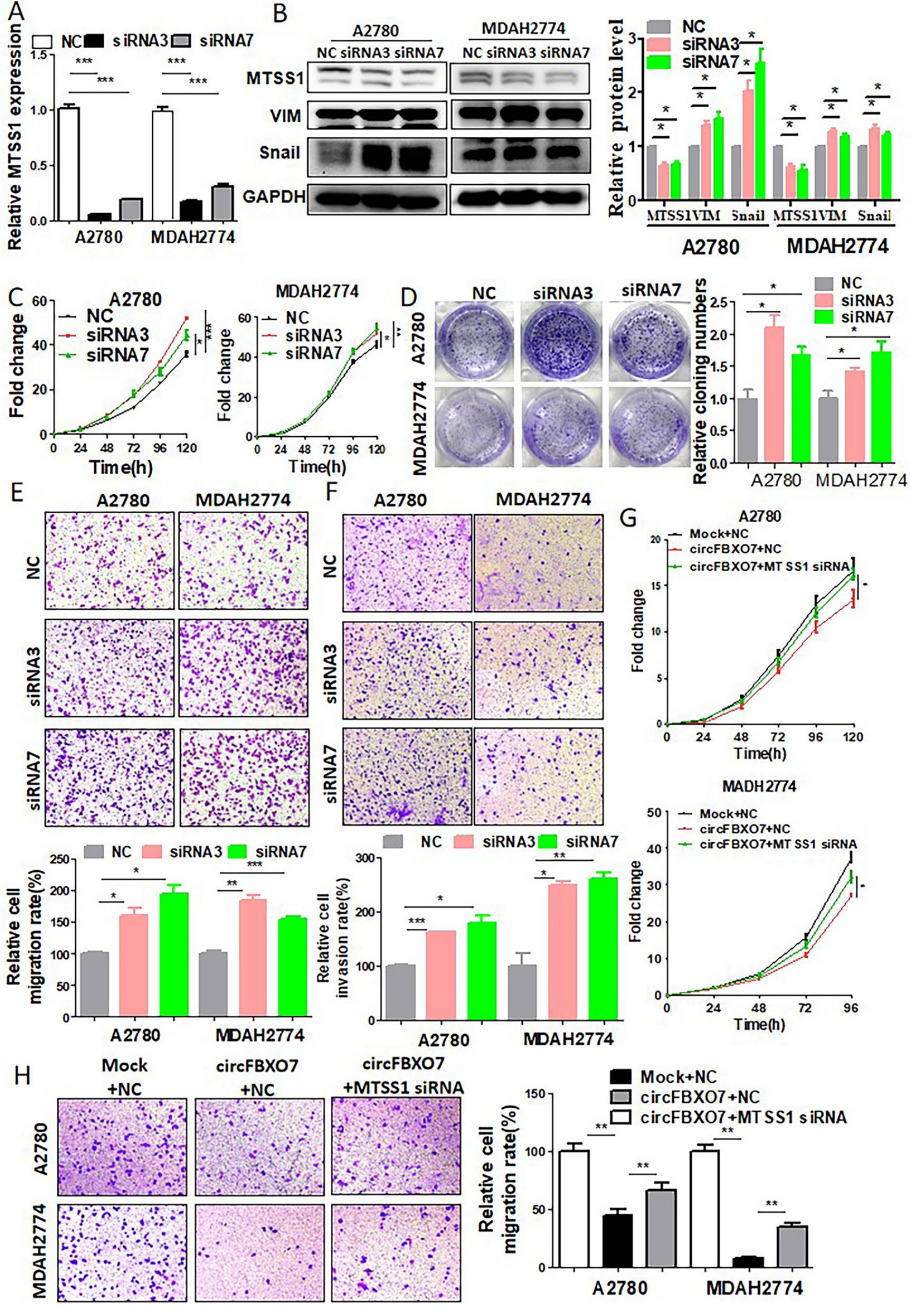
MTSS1 exerts tumor-suppressive effects in ovarian cancer cells. **A** qRT-PCR analysis of the expression of MTSS1 in A2780 and MDAH2774 cells transfected with MTSS1 siRNA or negative controls. **B** Western blot analysis of the expression of MTSS1, VIM, and Snail in A2780 and MDAH2774 cells transfected with MTSS1 siRNA or negative controls. **C** Growth curve of A2780 and MDAH2774 cells transfected with MTSS1 siRNA or negative controls as assessed using CCK-8 assays. **D** Proliferation of A2780 and MDAH2774 cells transfected with MTSS1 siRNA or negative controls as determined by colony formation assays. **E** Migration ability of A2780 and MDAH2774 cells transfected with MTSS1 siRNA or negative controls. **F** Invasion ability of A2780 and MDAH2774 cells transfected with MTSS1 siRNA or negative controls. **G** Growth curve of circFBXO7 overexpression A2780 and MDAH2774 cells transfected with MTSS1 siRNA or negative control siRNA. **H** Migration assays performed in circFBXO7 overexpression A2780 and MDAH2774 cells transfected with MTSS1 siRNA or negative control siRNA. *** *P* < 0.001, ** *P* < 0.01, * *P* < 0.05

MTSS1 has been characterized as a tumor suppressor in breast cancer [35] and chronic myeloid leukemia [36], however overexpression of MTSS1 has been observed in melanomas and hepatocellular carcinomas [37–39]. To explore the role of MTSS1 in ovarian cancer, we knocked down MTSS1 by siRNA and overexpressed it by overexpression plasmids in A2780 and MDAH2774 ovarian cancer cells (Fig. 5A, B, and Fig. S3A, B). Knockdown of MTSS1 promoted cell proliferation (Fig. 5C), colony formation (Fig. 5D), migration (Fig. 5E) and invasion (Fig. 5F). Conversely, overexpression of MTSS1 inhibited cell proliferation (Fig. S3C), colony formation (Fig. S3D) and migration (Fig. S3E). Furthermore, western blot showed that MTSS1 knockdown or overexpression increased or decreased the expressions of both SNAIL1 and vimentin, respectively (Fig. 5B and Fig. S3F), indicating that MTSS1 is involved in EMT.

As described above, circFBXO7 acts as a ceRNA for miR-96-5p to regulate the expression of MTSS1. MTSS1 in turn, is a target of miR-96-5p, and plays a tumor suppressive role in ovarian cancer. This suggests that circFBXO7 may regulate the occurrence and development of ovarian cancer through the circFBXO7/miR-96-5p/MTSS1 axis. To test this hypothesis, we knocked down MTSS1 by siRNA in the circFBXO7-overpressing A2780 and MDAH2774 cells. As a result, MTSS1 knockdown significantly rescued the decreased cell proliferation and migration caused by circFBXO7 overexpression (Fig. 5G and H). These results confirmed our hypothesis that circFBXO7 suppress the proliferation and migration of ovarian cancer cells through sponging miR-96-5p to regulate MTSS1.

### circFBXO7 regulates ovarian cancer through the miR-96-5p/MTSS1/wnt/β-catenin signaling pathway

**Fig. 6:**
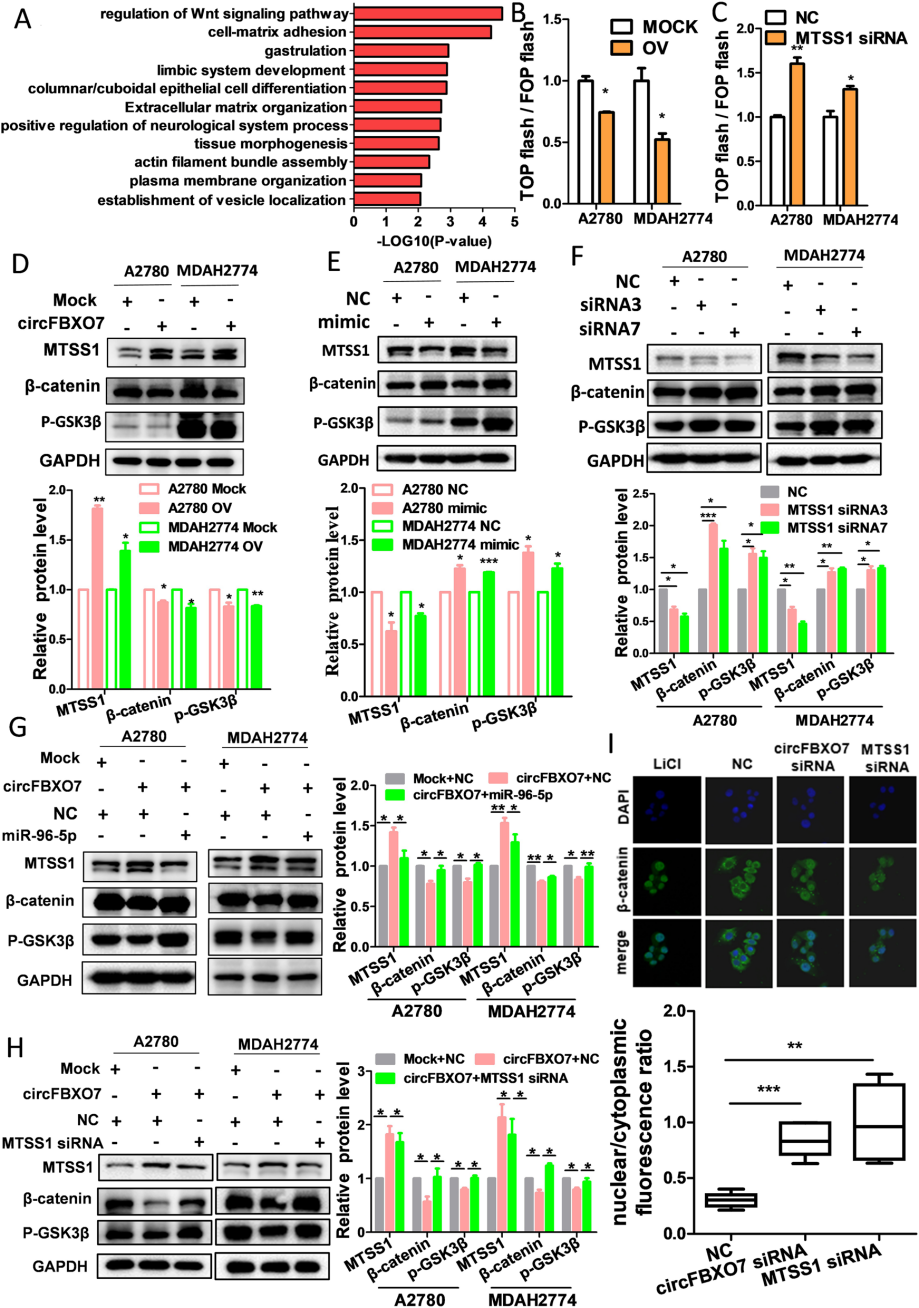
circFBXO7 regulates Wnt/β-catenin signaling pathway in ovarian cancer. **A** Enrichment analysis of signaling pathway after circFBXO7 overexpression using RNA-seq data. **B** Topflash/Fopflash assays performed in MDAH2774 and A2780 cells with circFBXO7 overexpression plasmid transfection. **C** Topflash/Fopflash assays performed in MDAH2774 and A2780 cells with MTSS1 siRNA transfection. **D** Western blot analysis of the expression of Wnt/β-catenin and p-GSK3β in A2780 and MDAH2774 cells transfected with circFBXO7 or mock plasmids, miR-96-5p mimics or negative control mimics**,** MTSS1 siRNA or negative controls. **E** Western blot analysis of the expression of Wnt/β-catenin and p-GSK3β in circFBXO7 overexpression A2780 and MDAH2774 cells transfected with miR-96-5p mimics or negative control mimics. **F** Western blot analysis of the expression of Wnt/β-catenin and p-GSK3β in circFBXO7 overexpression A2780 and MDAH2774 cells transfected with MTSS1 siRNA or negative controls. **G** Western blot analysis of the expression of Wnt/β-catenin and p-GSK3β in circFBXO7 overexpression A2780 and MDAH2774 cells transfected with miR-96-5p mimics or negative control mimics. **H** Western blot analysis of the expression of Wnt/β-catenin and p-GSK3β in circFBXO7 overexpression A2780 and MDAH2774 cells transfected with MTSS1 siRNA or negative control siRNA. **i** Representative images of IF micrographs of the subcellular localization and expression of β-catenin (green) in MDAH2774 cells transfected with circFBXO7 siRNA, MTSS1 siRNA, or negative control siRNA. Nuclei were counterstained with DAPI (blue). LiCl (Lithium chloride) is a Wnt activator which acts directly on GSK3β. *** *P* < 0.001, ** *P* < 0.01, * *P* < 0.05

To investigate the potential signaling pathways regulated by circFBXO7 in ovarian cancer cells, RNA-seq analysis was performed on circFBXO7 overexpressing and control cells. Differentially expressed genes (DEGs) between circFBXO7 overexpressing and control cells were detected from these RNA-seq data (Fig. S4). Regulation of Wnt/β-catenin signaling pathway was the most significant pathway that was enriched for DEGs upon overexpression of circFBXO7 (Fig. 6A). A growing body of literature has reported that WNTs, and their downstream effectors, regulate various important processes associated with cancer including tumor initiation, tumor growth, cell senescence, cell death, differentiation, and metastasis [40–42]. TOP/FOP-Flash reporter assays are often used to detect activity of the Wnt/β-catenin signaling pathway. Thus, we co-transfected circFBXO7 overexpression vector together with TOP flash or the control FOP flash, and MTSS1 siRNA together with TOP flash or the control FOP flash in A2780 and MDAH2774 cells, respectively. As a result, overexpression of circFBXO7 inhibited Wnt/β-catenin signaling activation, and knockdown of MTSS1 could activated Wnt/β-catenin signaling pathways in both cell lines (Fig. 6B and C). Western blot showed that overexpressing circFBXO7 or MTSS1 could consistently downregulate the expression of β-catenin and p-GSK3β (Fig. 6D and Fig. S5A), while miR-96-5p mimics or MTSS1 knockdown led to the upregulation of β-catenin and p-GSK3β (Fig. 6E and F). Furthermore, circFBXO7 knockdown also increased the protein level of β-catenin and p-GSK3β (Fig. S5B). In agreement with the western blot results, confocal immunostaining results for β-catenin showed that more β-catenin translocated to the nucleus upon knockdown of circFBXO7 or MTSS1 (Fig. 6I).

**Fig. 7:**
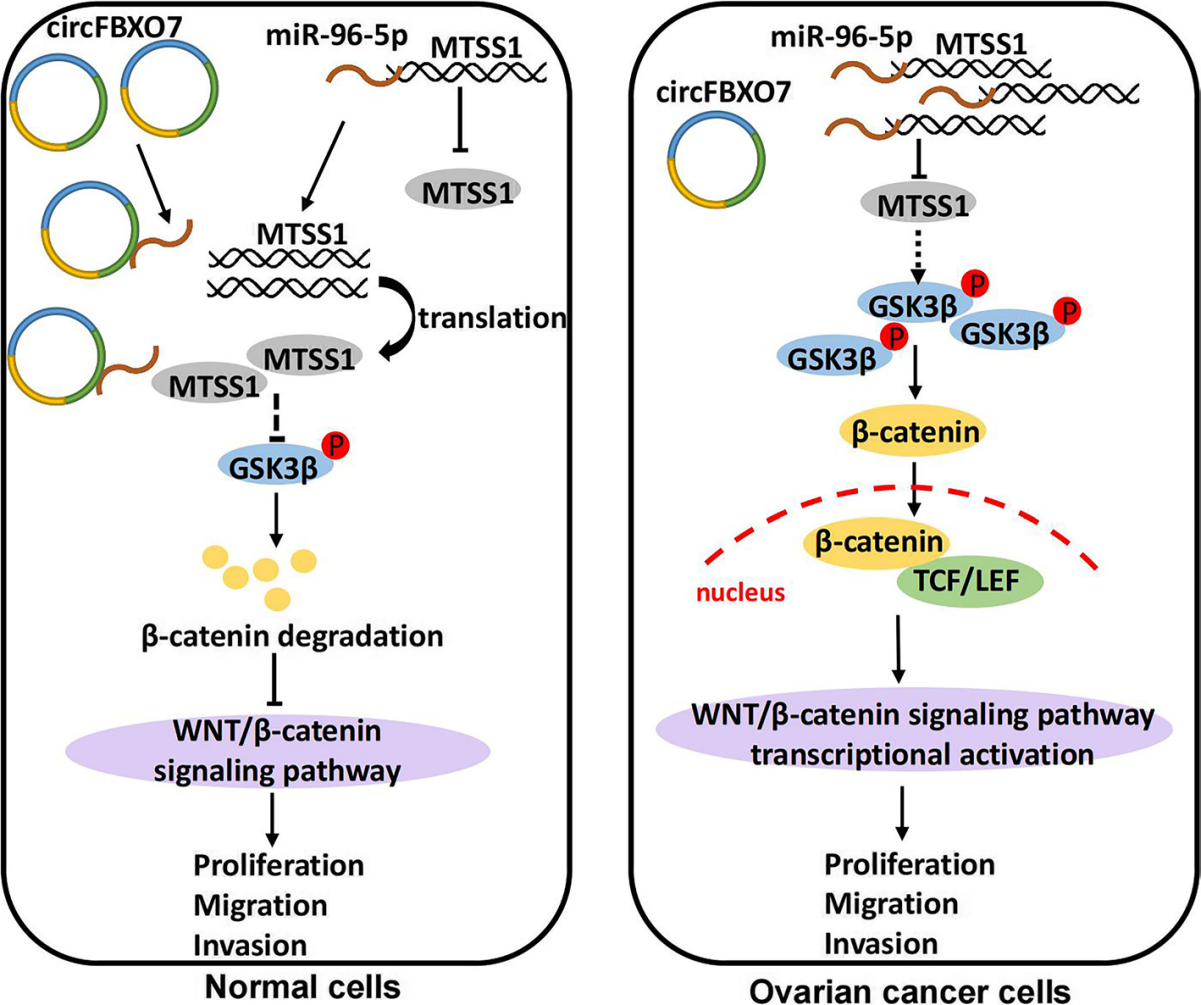
Potential molecular mechanism of circFBXO7 in ovarian cancer. circFBXO7 regulates ovarian cancer cell proliferation, migration and invasion through the miR-96-5p/MTSS1/wnt/β-catenin signaling pathway

Then, we carried out rescue experiments to further evaluate the regulatory effect of the circFBXO7/miR-96-5p/MTSS1 axis on the Wnt/β-catenin signaling pathway. As expected, miR-96-5p mimics or MTSS1 siRNA could rescue the drop in β-catenin and p-GSK3β protein levels due to circFBXO7 overexpression (Fig. 6G and H). Lithium chloride (LiCl) is a potent agonist of the Wnt signaling pathway which can inhibit GSK3β activity and lead to stabilization of β-catenin, thereby promoting the Wnt signaling pathway [43, 44]. Therefore, LiCl was used to counteract MTSS1 effects on the Wnt signaling in our rescue experiment. We found that LiCl could restore β-catenin and p-GSK3β protein levels that were dropped by MTSS1 overexpression (Fig. S5C). Consistently, LiCl could also enhance nuclear translocation of β-catenin that were blocked by MTSS1 overexpression (Fig. S5D). Taken together, our data revealed a working model that circFBXO7 inactivates Wnt/β-catenin signaling pathway by regulating the miR-96-5p/MTSS1 axis in ovarian cancer cells (Fig. 7).

## Discussion

A growing body of evidence suggests that circRNAs play an important role in the development and progression of cancer. In this study, we performed deep rRNA-depleted RNA-seq for 27 tumor tissues from ovarian cancer patients and 26 normal ovarian tissues from patients with gynaecological diseases and identified circFBXO7 as one of the most significantly downregulated circRNAs in ovarian cancer. CircFBXO7 is a novel circRNA and its clinical significance and functional role in ovarian cancer has not yet been explored. Using BaseScope assays, an RNA in situ hybridization technique that utilizes z-type probes to increase detection sensitivity while reducing background noise by signal amplification [45, 46], we assessed the expression of circFBXO7 in FFPE tissues from ovarian cancer patients with long-term follow-up. BaseScope assay results showed that although there was no significant difference in circFBXO7 expression between high-grade and low-grade ovarian tumors (Fig. S6), low circFBXO7 expression was highly predictive of a poor prognosis, suggesting circFBXO7 as a potential prognostic biomarker for ovarian cancer.

Both in vitro and in vivo assays demonstrated that the overexpression of circFBXO7 inhibits the proliferation, migration, and invasion of ovarian cancer cells, and decreases both EMT and its corresponding promotion of tumor metastasis. Conversely, the knockdown of circFBXO7 has the opposite effect. Increasing evidence suggests that circRNA can function as transcriptional regulators [47], miRNA sponges [6], protein decoys [7] or protein translation templates [8] to regulate gene expression at transcriptional or post-transcriptional levels where circRNAs in different subcellular compartments can execute different regulatory functions [48] . In this study, we found that circFBXO7 is mainly localized in the cytoplasm of ovarian cancer cells and possesses a complementary sequence to the miR-96-5p seed region. Luciferase reporter, FISH and RIP assays consistently revealed that circFBXO7 shows direct binding to miR-96-5p which itself has been suggested as an oncogene active in almost all studied cancers (e.g., ovarian, lung, head and neck, and breast cancer) [28–31]. In addition, transfecting miR-96-5p mimics significantly rescued the inhibitory effects of circFBXO7 overexpression on cell proliferation and migration. All such results indicated that circFBXO7 and miR-96-5p physically bind and interact with each other and are involved in regulating the proliferation, migration and invasion of ovarian cancer cells.

Three miRNA databases were used to find the common target genes of miR-96-5p. MTSS1 was identified as the strongest target gene as directly regulated by miR-96-5p. It has been reported that miR-96-5p may bind to MTSS1. MiR-96 was upregulated in cholangiocarcinoma and positively associated with poor prognosis; its upregulation may promote cholangiocarcinoma cell proliferation, migration and invasion by targeting MTSS1 [49]. miR-96 has also been reported to be increased in breast cancer; its overexpression may suppress breast cancer cell migration by downregulating the expression of MTSS1 [50]. MTSS1 had been previously considered a potential tumor suppressor in various cancers [35, 36, 51], but as an oncogene in hepatocellular carcinomas [37]. Meanwhile, some studies have shown that MTSS1 may be a scaffold protein that interacts with actin-associated proteins to modulate lamellipodia formation [52–55], or inhibit the Src-dependent phosphorylation of cortactin to promote dermal cilia regulation [56]. In our study, depletion of MTSS1 promoted cell proliferation and metastasis while overexpression of MTSS1 exerted the opposite role. Further rescue experiments revealed that circFBXO7 can modulate MTSS1 by competitively sponging miR-96-5p to inhibit ovarian cancer cell proliferation and metastasis.

RNA-seq analysis of circFBXO7 overexpression cells revealed Wnt signaling as one of the top targeted pathways upon circFBXO7 overexpression in ovarian cancer cells. The Wnt signaling pathway plays an important role in various cancers where β-catenin, as the core molecule in the Wnt signaling pathway, is involved in the transcriptional regulation of many genes [57]. Abnormal activation of Wnt signaling pathway is noted as one of the main contributors to the occurrence and development of many cancers, with the excessive accumulation of β-catenin as one of the key inducing factors for abnormal activation of Wnt signaling pathway [58]. GSK3β is the upstream regulator of β-catenin [59]. When Wnt signal is activated it can increase the phosphorylation of GSK3β, decrease the activity of GSK3β, and subsequently promote the accumulation of β-catenin. It could be that upregulation of circFBXO7 acts to reduce the protein levels of β-catenin and p-GSK3β, whilst the transfection with miR-96-5p or knocking down of MTSS1 results in their increase. Furthermore, transfection with miR-96-5p mimics or MTSS1 siRNA in ovarian cancer cells with circFBXO7 overexpression was able to dramatically rescue the suppressed Wnt signaling activation caused by overexpression of circFBXO7. Similarly, the suppression of β-catenin and p-GSK3β protein levels caused by MTSS1 overexpression could be rescued by the addition of LiCl.

It is worth noting that an interesting study has just been published recently, that is, MTSS1/Src2 shows the stimulatory effect of the Wnt/β-catenin on mouse osteoblast differentiation and bone homeostasis [60]. MTSS1 is a well-recognized antagonist of Src family of tyrosine kinases in multiple cellular processes [56, 61]. It has been reported that Src exhibits either stimulatory or inhibitory effects on canonical Wnt signaling, depending on the cellular context. In tumor cells, Src promotes tumor progression at least in part by enhancing Wnt signaling [62, 63]. Src functions as a positive regulator of β-catenin by docking to and being activated by Dishevelled-2 [64], resulting in increased β-catenin translocation into the nucleus [65]. In contrast, Src exhibits inhibitory effects on Wnt signaling in non-tumor cells, such as mouse osteoblast [60] and embryonic fibroblasts [66]. For example, Src phosphorylates LRP6 at conserved tyrosine residues, resulting in LRP6 removal from the cell surface and disruption of LRP6 signalosome formation, ultimately blocking Wnt signaling [66]. These results appear to explain why MTSS1 plays an apparently opposite role in ovarian cancer cells compared to non-cancer cells.

Several study caveats should be also acknowledged. Firstly, due to the polyA-enriched RNA-seq preparation protocol used in TCGA projects, TCGA has only linear FBXO7 expression data, but no circFBXO7 expression. Therefore, to evaluate the prognostic value of circFBXO7, we collected FFPE tissues from patients diagnosed with ovarian cancer in the Women’s Hospital of Zhejiang University from 2006 to 2012, with complete clinical and follow-up information. Approximately 30% of patients were collected after 2006 in the TCGA cohort, compared to 100% in our cohort. Due to improved diagnostic methods, patients in our cohort were diagnosed at an earlier age and had earlier tumor stage and better clinical outcome compared to the TCGA cohort. Considering that the FXBO7 expression was not significantly different between low-grade and high-grade ovarian cancer samples (*P* = 0.39, Student’s t-test) (Fig. S6), the results of survival analysis of FXBO7 in the TCGA dataset were not biased by tumor stage difference and remained informative. These results also indicated that the expression change of circFBXO7 likely occurred at a relatively early stage. Secondly, under circFBXO7/MTSS1 inhibition, the increase in phosphorylated GSK3β was generally less pronounced than nuclear translocated β-catenin. GSK3β is the upstream regulator of β-catenin. Simulated by Wnt signaling, GSK3β can increase the phosphorylation of GSK3β and reduce the activity of GSK3β, resulting in the release of β-catenin from β-catenin destruction complex [67]. In addition to the partial regulation of β-catenin by phosphorylated GSK3β, there may be other downstream factors regulated by circFBXO7/MTSS1 to regulate β-catenin expression or promote β-catenin nuclear translocation in ovarian cancer cells. This warrants further investigation in the future.

## Conclusions

In summary, circFBXO7 is downregulated in ovarian cancer. Patients with lower circFBXO7 expression have worse prognosis. circFBXO7 acts as a bone fide tumor suppressor in ovarian cancer. Downregulation of circFBXO7 releases its spongy miR-96-5p that then induces the degradation of MTSS1. The circFBXO7/miR-96-5p/MTSS1 axis is revealed as an important regulator in the Wnt signaling pathway. Targeting this axis may be a promising strategy for ovarian cancer treatment.

## Supplementary Information

**Additional file 1: Table S1.** Demographic and clinical information of ovarian cancer patients used in this study. **Table S2.** Quality control metrics of rRNA-depleted RNA-seq libraries in this study. **Table S3.** Sequences of primers and siRNAs used in this study. **Table S4.** Quality control metrics of ployA-enriched RNA-seq libraries in this study. **Table S5.** Information of antibodies used in the study. **Figure S1.** Genomic information of circFBXO7. (A) circBase annotation of circFBXO7 (ID: hsa_circ_0001222). (B) Sequence of full-length circFBXO7 from Sanger sequencing. **Figure S2.** circFBXO7 exerts tumor suppressive effects in ovarian cancer cells. (A) qRT-PCR analysis of circFBXO7 and linear-FBXO7 expression in SKOV3 and OV90 cells transfected with circFBXO7 siRNA and negative control siRNA. (B) Growth curve of SKOV3 and OV90 cells transfected with circFBXO7 siRNA and negative control siRNA, as assessed by CCK-8 assays. (C) Migration ability of SKOV3 and OV90 cells transfected with circFBXO7 siRNA and negative control siRNAs, as assessed by transwell assays. (D) Invasion ability of SKOV3 and OV90 cells transfected with circFBXO7 siRNA and negative control siRNAs, as assessed by transwell assays. **Figure S3.** MTSS1 inhibits ovarian cancer cell proliferation and migration. (A) qRT-PCR analysis of MTSS1 expression in A2780 and MDAH2774 cells transfected with MTSS1 overexpression and mock plasmids. (B) Western blot analysis of the expression of MTSS1 in A2780 and MDAH2774 cells transfected with MTSS1 overexpression and mock plasmids. (C) Growth curve of A2780 and MDAH2774 transfected with MTSS1 overexpression and mock plasmids, as assessed by CCK-8 assays. (D) Proliferation of A2780 and MDAH2774 cells transfected with MTSS1 overexpression and mock plasmids, as determined by colony formation assay. (E) Migration ability of A2780 and MDAH2774 cells transfected with MTSS1 overexpression and mock plasmids. (F) Western blot analysis of the expression of VIM and snail in A2780 and MDAH2774 cells transfected with MTSS1 siRNA and negative control siRNA. Student’s two-sided t tests were used. *** *P* < 0.001, ** *P* < 0.01, * *P* < 0.05. **Figure S4.** Heat map of differentially expressed genes between circFBXO7-overexpressing and control ovarian cancer cells. **Figure S5.** circFBXO7/MTSS1 is involved in the regulation of Wnt/β-catenin signaling. (A) Western blot analysis of the expression of wnt/β-catenin and p-GSK3β in A2780 and MDAH2774 cells transfected with MTSS1 overexpression and mock plasmids. (B) Western blot analysis of the expression of β-catenin and p-GSK3β in A2780 and MDAH2774 cells transfected with circFBXO7 siRNA or negative controls. (C) Western blot analysis of the expression of β-catenin and p-GSK3β in MTSS1 overexpression A2780 and MDAH2774 cells transfected with LiCl, NaCl, or mock plasmids. (D) Representative images of IF micrographs of the subcellular localization and expression of β-catenin (green) in MTSS1 overexpression MDAH2774 cells transfected with LiCl. Nuclei were counterstained with DAPI (blue). Student’s two-sided t tests were used. *** *P* < 0.001, ** *P* < 0.01, * *P* < 0.05. **Figure S6.** circFBXO7 and FBXO7 expression in low- and high-grade ovarian tumors. (A) CircFBXO7 expression in our FFPE cohort. (B) FBXO7 expression in the TCGA cohort.

## Data Availability

The raw sequence data are deposited in the Genome Sequence Archive (GSA) under accession HRA001962 (http://bigd.big.ac.cn/gsa-human). All other data generated during this study are included in this published article and its supplementary files.

## Abbreviations

CCK8: a Cell Counting Kit-8
CircRNA: Circular RNA
DEGs: Differentially Expressed Genes
EMT: Epithelial-mesenchymal Transitions
FFPE: Formalin Fixation and Paraffin Embedding
GSK3β: Glycogen Synthase Kinase 3 beta
LiCl: Lithium Chloride
miRNA: microRNA
MTSS1: Metastasis Suppressor 1
PVDF: Polyvinylidene Fluoride
RBPs: RNA Binding Proteins
RIP: RNA Immunoprecipitation
RNA-seq: Genome-wide Transcriptome Analysis
SDS-PAGE: Sodium Dodecyl Sulfate-polyacrylamide Gel Electrophoresis
siRNA: Small Interference RNA
β-catenin: Catenin Beta 1

## References

[1] Chen LL (2016). The biogenesis and emerging roles of circular RNAs. Nat Rev Mol Cell Biol.

[2] Wilusz JE, Sharp PA (2013). Molecular biology. A circuitous route to noncoding RNA. Science.

[3] Arnaiz E, Sole C, Manterola L, Iparraguirre L, Otaegui D, Lawrie CH (2019). CircRNAs and cancer: biomarkers and master regulators. Semin Cancer Biol.

[4] Ashwal-Fluss R, Meyer M, Pamudurti NR, Ivanov A, Bartok O, Hanan M, Evantal N, Memczak S, Rajewsky N, Kadener S (2014). circRNA biogenesis competes with pre-mRNA splicing. Mol Cell.

[5] Shen T, Han M, Wei G, Ni T (2015). An intriguing RNA species--perspectives of circularized RNA. Protein Cell.

[6] Thomson DW, Dinger ME (2016). Endogenous microRNA sponges: evidence and controversy. Nat Rev Genet.

[7] Zhou WY, Cai ZR, Liu J, Wang DS, Ju HQ, Xu RH (2020). Circular RNA: metabolism, functions and interactions with proteins. Mol Cancer.

[8] Pamudurti NR, Bartok O, Jens M, Ashwal-Fluss R, Stottmeister C, Ruhe L, Hanan M, Wyler E, Perez-Hernandez D, Ramberger E (2017). Translation of CircRNAs. Mol Cell.

[9] Li Q, Pan X, Zhu D, Deng Z, Jiang R, Wang X (2019). Circular RNA MAT2B promotes glycolysis and malignancy of hepatocellular carcinoma through the miR-338-3p/PKM2 Axis under hypoxic stress. Hepatology.

[10] Cheng Z, Yu C, Cui S, Wang H, Jin H, Wang C, Li B, Qin M, Yang C, He J (2019). circTP63 functions as a ceRNA to promote lung squamous cell carcinoma progression by upregulating FOXM1. Nat Commun.

[11] Yang F, Hu A, Li D, Wang J, Guo Y, Liu Y, Li H, Chen Y, Wang X, Huang K (2019). Circ-HuR suppresses HuR expression and gastric cancer progression by inhibiting CNBP transactivation. Mol Cancer.

[12] Legnini I, Di Timoteo G, Rossi F, Morlando M, Briganti F, Sthandier O, Fatica A, Santini T, Andronache A, Wade M (2017). Circ-ZNF609 is a circular RNA that can be translated and functions in Myogenesis. Mol Cell.

[13] Wu X, Xiao S, Zhang M, Yang L, Zhong J, Li B, Li F, Xia X, Li X, Zhou H (2021). A novel protein encoded by circular SMO RNA is essential for hedgehog signaling activation and glioblastoma tumorigenicity. Genome Biol.

[14] Meng S, Zhou H, Feng Z, Xu Z, Tang Y, Li P, Wu M (2017). CircRNA: functions and properties of a novel potential biomarker for cancer. Mol Cancer.

[15] Vo JN, Cieslik M, Zhang Y, Shukla S, Xiao L, Zhang Y, Wu YM, Dhanasekaran SM, Engelke CG, Cao X (2019). The landscape of circular RNA in Cancer. Cell.

[16] Siegel RL, Miller KD, Jemal A (2020). Cancer statistics, 2020. CA Cancer J Clin.

[17] Bast RC, Hennessy B, Mills GB (2009). The biology of ovarian cancer: new opportunities for translation. Nat Rev Cancer.

[18] Bounoutas A, Zheng Q, Nonet ML, Chalfie M (2009). Mec-15 encodes an F-box protein required for touch receptor neuron mechanosensation, synapse formation and development. Genetics.

[19] Laman H, Funes JM, Ye H, Henderson S, Galinanes-Garcia L, Hara E, Knowles P, McDonald N, Boshoff C (2005). Transforming activity of Fbxo7 is mediated specifically through regulation of cyclin D/cdk6. EMBO J.

[20] Kim WY, Geng R, Somers DE (2003). Circadian phase-specific degradation of the F-box protein ZTL is mediated by the proteasome. Proc Natl Acad Sci U S A.

[21] Conedera S, Apaydin H, Li Y, Yoshino H, Ikeda A, Matsushima T, Funayama M, Nishioka K, Hattori N (2016). FBXO7 mutations in Parkinson's disease and multiple system atrophy. Neurobiol Aging.

[22] Kim D, Pertea G, Trapnell C, Pimentel H, Kelley R, Salzberg SL (2013). TopHat2: accurate alignment of transcriptomes in the presence of insertions, deletions and gene fusions. Genome Biol.

[23] Zhang XO, Dong R, Zhang Y, Zhang JL, Luo Z, Zhang J, Chen LL, Yang L (2016). Diverse alternative back-splicing and alternative splicing landscape of circular RNAs. Genome Res.

[24] Zhang L, Zhou Q, Qiu Q, Hou L, Wu M, Li J, Li X, Lu B, Cheng X, Liu P (2019). CircPLEKHM3 acts as a tumor suppressor through regulation of the miR-9/BRCA1/DNAJB6/KLF4/AKT1 axis in ovarian cancer. Mol Cancer.

[25] Kovaka S, Zimin AV, Pertea GM, Razaghi R, Salzberg SL, Pertea M (2019). Transcriptome assembly from long-read RNA-seq alignments with StringTie2. Genome Biol.

[26] Trapnell C, Hendrickson DG, Sauvageau M, Goff L, Rinn JL, Pachter L (2013). Differential analysis of gene regulation at transcript resolution with RNA-seq. Nat Biotechnol.

[27] Cancer genome atlas research N (2011). Integrated genomic analyses of ovarian carcinoma. Nature.

[28] Liu B, Zhang J, Yang D (2019). miR-96-5p promotes the proliferation and migration of ovarian cancer cells by suppressing Caveolae1. J Ovarian Res.

[29] Wei S, Zheng Y, Jiang Y, Li X, Geng J, Shen Y, Li Q, Wang X, Zhao C, Chen Y (2019). The circRNA circPTPRA suppresses epithelial-mesenchymal transitioning and metastasis of NSCLC cells by sponging miR-96-5p. EBioMedicine.

[30] Vahabi M, Pulito C, Sacconi A, Donzelli S, D'Andrea M, Manciocco V, Pellini R, Paci P, Sanguineti G, Strigari L (2019). miR-96-5p targets PTEN expression affecting radio-chemosensitivity of HNSCC cells. J Exp Clin Cancer Res.

[31] Matamala N, Vargas MT, Gonzalez-Campora R, Minambres R, Arias JI, Menendez P, Andres-Leon E, Gomez-Lopez G, Yanowsky K, Calvete-Candenas J (2015). Tumor microRNA expression profiling identifies circulating microRNAs for early breast cancer detection. Clin Chem.

[32] Agarwal V, Bell GW, Nam JW, Bartel DP. Predicting effective microRNA target sites in mammalian mRNAs. Elife. 2015;4:e05005. 10.7554/eLife.05005 PMC4532895 26267216

[33] Chen Y, Wang X (2020). miRDB: an online database for prediction of functional microRNA targets. Nucleic Acids Res.

[34] Huang HY, Lin YC, Li J, Huang KY, Shrestha S, Hong HC, Tang Y, Chen YG, Jin CN, Yu Y (2020). miRTarBase 2020: updates to the experimentally validated microRNA-target interaction database. Nucleic Acids Res.

[35] Parr C, Jiang WG (2009). Metastasis suppressor 1 (MTSS1) demonstrates prognostic value and anti-metastatic properties in breast cancer. Eur J Cancer.

[36] Schemionek M, Herrmann O, Reher MM, Chatain N, Schubert C, Costa IG, Hanzelmann S, Gusmao EG, Kintsler S, Braunschweig T (2016). Mtss1 is a critical epigenetically regulated tumor suppressor in CML. Leukemia.

[37] Ma S, Guan XY, Lee TK, Chan KW (2007). Clinicopathological significance of missing in metastasis B expression in hepatocellular carcinoma. Hum Pathol.

[38] Huang XY, Huang ZL, Xu B, Chen Z, Re TJ, Zheng Q, Tang ZY, Huang XY (2016). Elevated MTSS1 expression associated with metastasis and poor prognosis of residual hepatitis B-related hepatocellular carcinoma. J Exp Clin Cancer Res.

[39] Mertz KD, Pathria G, Wagner C, Saarikangas J, Sboner A, Romanov J, Gschaider M, Lenz F, Neumann F, Schreiner W (2014). MTSS1 is a metastasis driver in a subset of human melanomas. Nat Commun.

[40] Anastas JN, Moon RT (2013). WNT signalling pathways as therapeutic targets in cancer. Nat Rev Cancer.

[41] Nusse R, Clevers H (2017). Wnt/beta-catenin signaling, disease, and emerging therapeutic modalities. Cell.

[42] Klaus A, Birchmeier W (2008). Wnt signalling and its impact on development and cancer. Nat Rev Cancer.

[43] Hedgepeth CM, Conrad LJ, Zhang J, Huang HC, Lee VM, Klein PS (1997). Activation of the Wnt signaling pathway: a molecular mechanism for lithium action. Dev Biol.

[44] Herath NI, Rocques N, Garancher A, Eychene A, Pouponnot C (2014). GSK3-mediated MAF phosphorylation in multiple myeloma as a potential therapeutic target. Blood Cancer J.

[45] Baker AM, Huang W, Wang XM, Jansen M, Ma XJ, Kim J, Anderson CM, Wu X, Pan L, Su N (1998). Robust RNA-based in situ mutation detection delineates colorectal cancer subclonal evolution. Nat Commun.

[46] Sengal AT, Patch AM, Snell CE, Smith DS, Leung SCY, Talhouk A, Williams ED, McAlpine JN, Pollock PM (2020). FGFR2c Mesenchymal isoform expression is associated with poor prognosis and further refines risk stratification within endometrial Cancer molecular subtypes. Clin Cancer Res.

[47] Li Z, Huang C, Bao C, Chen L, Lin M, Wang X, Zhong G, Yu B, Hu W, Dai L (2015). Exon-intron circular RNAs regulate transcription in the nucleus. Nat Struct Mol Biol.

[48] Wilusz JE (2018). A 360 degrees view of circular RNAs: from biogenesis to functions. Wiley Interdiscip Rev RNA.

[49] Yin X, Chai Z, Sun X, Chen J, Wu X, Yang L, Zhou X, Liu F (2020). Overexpression of microRNA-96 is associated with poor prognosis and promotes proliferation, migration and invasion in cholangiocarcinoma cells via MTSS1. Exp Ther Med.

[50] Xie W, Sun F, Chen L, Cao X (2018). miR-96 promotes breast cancer metastasis by suppressing MTSS1. Oncol Lett.

[51] Liu R, Martin TA, Jordan NJ, Ruge F, Ye L, Jiang WG (2015). Metastasis suppressor 1 expression in human ovarian cancer: the impact on cellular migration and metastasis. Int J Oncol.

[52] Mattila PK, Pykalainen A, Saarikangas J, Paavilainen VO, Vihinen H, Jokitalo E, Lappalainen P (2007). Missing-in-metastasis and IRSp53 deform PI(4,5)P2-rich membranes by an inverse BAR domain-like mechanism. J Cell Biol.

[53] Kawabata Galbraith K, Fujishima K, Mizuno H, Lee SJ, Uemura T, Sakimura K, Mishina M, Watanabe N, Kengaku M (2018). MTSS1 regulation of actin-nucleating Formin DAAM1 in dendritic Filopodia determines final dendritic configuration of Purkinje cells. Cell Rep.

[54] Bompard G, Sharp SJ, Freiss G, Machesky LM (2005). Involvement of Rac in actin cytoskeleton rearrangements induced by MIM-B. J Cell Sci.

[55] Lee SH, Kerff F, Chereau D, Ferron F, Klug A, Dominguez R (2007). Structural basis for the actin-binding function of missing-in-metastasis. Structure.

[56] Bershteyn M, Atwood SX, Woo WM, Li M, Oro AE (2010). MIM and cortactin antagonism regulates ciliogenesis and hedgehog signaling. Dev Cell.

[57] Li VS, Ng SS, Boersema PJ, Low TY, Karthaus WR, Gerlach JP, Mohammed S, Heck AJ, Maurice MM, Mahmoudi T, Clevers H (2012). Wnt signaling through inhibition of beta-catenin degradation in an intact Axin1 complex. Cell.

[58] Cui C, Zhou X, Zhang W, Qu Y, Ke X (2018). Is beta-catenin a Druggable target for Cancer therapy?. Trends Biochem Sci.

[59] Soutto M, Peng D, Katsha A, Chen Z, Piazuelo MB, Washington MK, Belkhiri A, Correa P, El-Rifai W (2015). Activation of beta-catenin signalling by TFF1 loss promotes cell proliferation and gastric tumorigenesis. Gut.

[60] Chen M, Shan L, Gan Y, Tian L, Zhou J, Zhu E, Yuan H, Li X, Wang B (2022). Metastasis suppressor 1 controls osteoblast differentiation and bone homeostasis through regulating Src-Wnt/beta-catenin signaling. Cell Mol Life Sci.

[61] Quinones GA, Jin J, Oro AE (2010). I-BAR protein antagonism of endocytosis mediates directional sensing during guided cell migration. J Cell Biol.

[62] Haraguchi K, Nishida A, Ishidate T, Akiyama T (2004). Activation of beta-catenin-TCF-mediated transcription by non-receptor tyrosine kinase v-Src. Biochem Biophys Res Commun.

[63] Karni R, Gus Y, Dor Y, Meyuhas O, Levitzki A (2005). Active Src elevates the expression of beta-catenin by enhancement of cap-dependent translation. Mol Cell Biol.

[64] Yokoyama N, Malbon CC (2009). Dishevelled-2 docks and activates Src in a Wnt-dependent manner. J Cell Sci.

[65] Condello S, Cao L, Matei D (2013). Tissue transglutaminase regulates beta-catenin signaling through a c-Src-dependent mechanism. FASEB J.

[66] Chen Q, Su Y, Wesslowski J, Hagemann AI, Ramialison M, Wittbrodt J, Scholpp S, Davidson G (2014). Tyrosine phosphorylation of LRP6 by Src and Fer inhibits Wnt/beta-catenin signalling. EMBO Rep.

[67] Wu D, Pan W (2010). GSK3: a multifaceted kinase in Wnt signaling. Trends Biochem Sci.

