# Supplementary material for: circFBXO7/miR-96-5p/MTSS1 axis is an important regulator in the Wnt signaling pathway in ovarian cancer
Source: Mol Cancer. 2022 Jun 29;21:137. doi: 10.1186/s12943-022-01611-y (PMC9241180; doi:10.1186/s12943-022-01611-y)
Supplement: Supplementary file 1 — Additional file 1: Table S1. Demographic and clinical information of ovarian cancer patients used in this study. Table S2. Quality control metrics of rRNA-depleted RNA-seq libraries in this study. Table S3. Sequences of primers and siRNAs used in this study. Table S4. Quality control metrics of ployA-enriched RNA-seq libraries in this study. Table S5. Information of antibodies used in the study. Figure S1. Genomic information of circFBXO7. (A) circBase annotation of circFBXO7 (ID: hsa_circ_0001222). (B) Sequence of full-length circFBXO7 from Sanger sequencing. Figure S2. circFBXO7 exerts tumor suppressive effects in ovarian cancer cells. (A) qRT-PCR analysis of circFBXO7 and linear-FBXO7 expression in SKOV3 and OV90 cells transfected with circFBXO7 siRNA and negative control siRNA. (B) Growth curve of SKOV3 and OV90 cells transfected with circFBXO7 siRNA and negative control siRNA, as assessed by CCK-8 assays. (C) Migration ability of SKOV3 and OV90 cells transfected with circFBXO7 siRNA and negative control siRNAs, as assessed by transwell assays. (D) Invasion ability of SKOV3 and OV90 cells transfected with circFBXO7 siRNA and negative control siRNAs, as assessed by transwell assays. Figure S3. MTSS1 inhibits ovarian cancer cell proliferation and migration. (A) qRT-PCR analysis of MTSS1 expression in A2780 and MDAH2774 cells transfected with MTSS1 overexpression and mock plasmids. (B) Western blot analysis of the expression of MTSS1 in A2780 and MDAH2774 cells transfected with MTSS1 overexpression and mock plasmids. (C) Growth curve of A2780 and MDAH2774 transfected with MTSS1 overexpression and mock plasmids, as assessed by CCK-8 assays. (D) Proliferation of A2780 and MDAH2774 cells transfected with MTSS1 overexpression and mock plasmids, as determined by colony formation assay. (E) Migration ability of A2780 and MDAH2774 cells transfected with MTSS1 overexpression and mock plasmids. (F) Western blot analysis of the expression of VIM and snail in A2780 and MDAH27 [file 12943_2022_1611_MOESM1_ESM.pdf]

Table S1 Demographic and clinical information of ovarian cancer patients used in this study

No.	Race	Age	Cancer type	Stage	Overall survival (follow up)		RFS (follow up)		Assays
					Status	Months	Status	Months	
1	Asian	39	Serous carcinoma	Ic	alive	138	no	138	Basescope
2	Asian	40	Endometriod tumor	Ic	alive	125	no	125	Basescope
3	Asian	36	Serous carcinoma	Ic	alive	125	no	125	Basescope
4	Asian	63	Serous carcinoma	IIb	alive	122	no	122	Basescope
5	Asian	37	Endometriod tumor	IIb	alive	129	no	129	Basescope
6	Asian	51	Serous carcinoma	IIb	alive	161	yes	84	Basescope
7	Asian	63	Serous carcinoma	IIIb	alive	169	no	169	Basescope
8	Asian	56	Serous carcinoma	IIc	alive	165	no	165	Basescope
9	Asian	42	Serous carcinoma	IIIc	alive	163	no	163	Basescope
10	Asian	55	Serous carcinoma	IIc	alive	112	yes	59	Basescope
11	Asian	72	Serous carcinoma	IIb	dead	137	yes	137	Basescope
12	Asian	50	Serous carcinoma	IIIc	alive	162	yes	24	Basescope
13	Asian	51	Endometriod tumor	IIb	alive	144	no	144	Basescope
14	Asian	71	Clear cell adenocarcinoma	Ic	alive	135	no	135	Basescope
15	Asian	49	Serous carcinoma	IIa	alive	128	no	128	Basescope
16	Asian	59	Serous carcinoma	IIIc	dead	35	yes	8	Basescope
17	Asian	50	Serous carcinoma	IIIc	dead	16	yes	6	Basescope
18	Asian	55	Serous carcinoma	IIIc	dead	16	yes	16	Basescope
19	Asian	60	Serous carcinoma	IIIb	dead	16	yes	14	Basescope
20	Asian	57	Serous carcinoma	IIIc	dead	15	yes	8	Basescope
21	Asian	37	Serous carcinoma	IIc	dead	64	yes	35	Basescope
22	Asian	47	Serous carcinoma	IIIc	dead	43	yes	24	Basescope
23	Asian	51	Serous carcinoma	IIIc	dead	33	yes	7	Basescope
24	Asian	49	Serous carcinoma	IIIc	dead	29	yes	21	Basescope
25	Asian	46	Serous carcinoma	IIIc	dead	41	yes	10	Basescope
26	Asian	53	Serous carcinoma	IIIc	dead	41	yes	18	Basescope
27	Asian	64	Serous carcinoma	IIIc	dead	50	yes	49	Basescope
28	Asian	52	Serous carcinoma	IIIc	dead	28	yes	15	Basescope
29	Asian	75	Serous carcinoma	IIIc	dead	33	yes	23	Basescope
30	Asian	55	Serous carcinoma	IIIc	dead	25	yes	9	Basescope
31	Asian	49	Serous carcinoma	IV	dead	14	yes	12	Basescope
32	Asian	49	Clear cell adenocarcinoma	Ic	alive	175	no	175	Basescope
33	Asian	53	Clear cell adenocarcinoma	Ic	alive	173	no	173	Basescope
34	Asian	55	Clear cell adenocarcinoma	Ic	alive	146	no	146	Basescope
35	Asian	55	Mixed epithelioma	IIIc	dead	35	yes	12	Basescope
36	Asian	56	Serous carcinoma	IIa	alive	139	no	139	Basescope
37	Asian	56	Serous carcinoma	IIIc	dead	19	yes	7	Basescope
38	Asian	52	Serous carcinoma	IIb	dead	65	yes	49	Basescope
39	Asian	54	Serous carcinoma	IIIc	alive	133	no	133	Basescope
40	Asian	55	Serous carcinoma	IIIc	alive	149	yes	3	Basescope
41	Asian	40	Serous carcinoma	IIIc	alive	158	no	158	Basescope
42	Asian	34	Serous carcinoma	IIIc	alive	165	no	165	Basescope
43	Asian	52	Serous carcinoma	IIb	alive	176	no	176	Basescope
44	Asian	63	Serous carcinoma	IIIc	dead	21	yes	16	Basescope
45	Asian	53	Serous carcinoma	IIIc	dead	79	yes	23	Basescope
46	Asian	64	Serous carcinoma	IIIb	alive	177	no	177	Basescope
47	Asian	50	Clear cell adenocarcinoma	Ic	alive	164	no	164	Basescope
48	Asian	51	Serous carcinoma	IIIc	dead	47	yes	20	Basescope
49	Asian	57	Serous carcinoma	IIIc	dead	25	yes	15	Basescope
50	Asian	22	Serous carcinoma	IIIc	dead	104	yes	104	Basescope
51	Asian	61	Clear cell adenocarcinoma	Ic	alive	126	no	126	Basescope
52	Asian	66	Endometriod tumor	IIc	dead	115	yes	91	Basescope
53	Asian	52	Clear cell adenocarcinoma	Ic	alive	134	no	134	Basescope

54	Asian	57	Serous carcinoma	IIIc	dead	7	yes	1	Basescope
55	Asian	64	Mixed epithelioma	IIa	dead	42	yes	4	Basescope
56	Asian	58	Serous carcinoma	IIIc	alive	126	no	126	Basescope
57	Asian	53	Serous carcinoma	IIIc	alive	125	no	125	Basescope
58	Asian	48	Mixed epithelioma	IIIb	dead	18	yes	5	Basescope
59	Asian	50	Serous carcinoma	Ib	alive	135	no	135	Basescope
60	Asian	38	Serous carcinoma	IIIc	dead	12	yes	10	Basescope
61	Asian	53	Serous carcinoma	IV(G3)	dead	6	yes	6	Basescope
62	Asian	49	Serous carcinoma	IIIb	alive	135	yes	41	Basescope
63	Asian	38	Serous carcinoma	IIIc	dead	33	yes	12	Basescope
64	Asian	66	Serous carcinoma	IIIc	dead	27	yes	17	Basescope
65	Asian	56	Serous carcinoma	IIIc	dead	18	yes	10	Basescope
66	Asian	64	Mixed epithelioma	IIa	dead	21	yes	17	Basescope
67	Asian	67	Serous carcinoma	IIc	dead	86	yes	71	Basescope
68	Asian	44	Serous carcinoma	IIIc	dead	22	yes	12	Basescope
69	Asian	60	Clear cell adenocarcinoma	IIIb	dead	38	yes	32	Basescope
70	Asian	54	Serous carcinoma	IIIc	dead	9	yes	9	Basescope
71	Asian	50	Serous carcinoma	IIIc	/	/	/	/	RNA-seq
72	Asian	55	Serous carcinoma	IIIc	/	/	/	/	RNA-seq
73	Asian	57	Serous carcinoma	IIb	/	/	/	/	RNA-seq
74	Asian	66	Serous carcinoma	IIIc	/	/	/	/	RNA-seq
75	Asian	46	Serous carcinoma	IIIA1	/	/	/	/	RNA-seq
76	Asian	60	Mixed epithelioma	IIIb	/	/	/	/	RNA-seq
77	Asian	54	Serous carcinoma	IIIc	/	/	/	/	RNA-seq
78	Asian	45	Serous carcinoma	IIIc	/	/	/	/	RNA-seq
79	Asian	63	Serous carcinoma	IIIc	/	/	/	/	RNA-seq
80	Asian	60	Serous carcinoma	IIIb	/	/	/	/	RNA-seq
81	Asian	46	Clear cell adenocarcinoma	IIIa2	/	/	/	/	RNA-seq
82	Asian	42	Clear cell adenocarcinoma	Ia	/	/	/	/	RNA-seq
83	Asian	53	Clear cell adenocarcinoma	IIb	/	/	/	/	RNA-seq
84	Asian	57	Serous carcinoma	IIb	/	/	/	/	RNA-seq
85	Asian	57	Serous carcinoma	IIb	/	/	/	/	RNA-seq
86	Asian	44	Clear cell adenocarcinoma	Ia	/	/	/	/	RNA-seq
87	Asian	58	Clear cell adenocarcinoma	Ic2	/	/	/	/	RNA-seq
88	Asian	64	Serous carcinoma	IIIC	/	/	/	/	RNA-seq
89	Asian	33	Serous carcinoma	IIIA1	/	/	/	/	RNA-seq
90	Asian	64	Serous carcinoma	IIIC	/	/	/	/	RNA-seq
91	Asian	44	Serous carcinoma	IIIA2	/	/	/	/	RNA-seq
92	Asian	71	Serous carcinoma	IIIb	/	/	/	/	RNA-seq
93	Asian	49	Serous carcinoma	IIIb	/	/	/	/	RNA-seq
94	Asian	52	Serous carcinoma	IIIc	/	/	/	/	RNA-seq
95	Asian	64	Serous carcinoma	Ivb	/	/	/	/	RNA-seq
96	Asian	46	Serous carcinoma	Ia	/	/	/	/	RNA-seq
97	Asian	57	Clear cell adenocarcinoma	IIIb	/	/	/	/	RNA-seq

Table S2 Quality control metrics of rRNA-depleted RNA-seq libraries in this study

Sample ID	Tissue	Yield (Gb)	# Reads	% of >= Q30 Bases	Mean Quality Score	% of mapping
NC10	Normal	20.0	143,066,598	96.27%	38.0	81.7
NC13	Normal	10.5	75,433,010	98.23%	39.4	80.7
NC14	Normal	21.9	156,582,656	96.13%	38.5	71.7
NC22	Normal	16.6	118,895,824	96.81%	38.2	82
NC24	Normal	17.0	121,857,740	95.95%	37.9	82.7
NC26	Normal	18.8	134,385,746	96.07%	37.9	81.3
NC28	Normal	16.9	121,161,462	94.95%	37.8	80.4
NC30	Normal	17.5	125,114,032	96.23%	38.0	86.3
NC32	Normal	18.0	129,062,864	96.21%	38.1	84
NC36	Normal	16.6	119,024,948	96.48%	38.2	84.3
NC38	Normal	16.7	119,884,830	96.02%	38.0	81.6
NC4	Normal	19.0	136,112,844	96.42%	38.6	85.4
NC40	Normal	18.0	128,941,858	95.90%	37.9	81.6
NC42	Normal	17.6	126,128,364	95.20%	37.6	73.8
NC44	Normal	18.0	129,058,384	95.38%	37.8	81.5
NC46	Normal	16.1	114,975,662	95.10%	37.7	80.1
NC48	Normal	16.9	120,994,368	95.81%	37.8	83.2
NC50	Normal	18.1	129,537,084	95.53%	37.8	82.8
NC52	Normal	18.2	130,031,908	95.91%	37.9	83.4
NC54	Normal	18.3	131,241,188	96.79%	38.3	85.9
NC55	Normal	19.8	141,608,594	93.79%	36.9	82.4
NC57	Normal	12.5	89,471,892	93.06%	36.6	79.2
NC6	Normal	18.8	134,536,734	96.32%	38.5	83.3
NC8	Normal	17.9	127,868,040	96.08%	38.0	84.2
OC4	Normal	11.3	80,596,116	96.70%	38.7	87.4
OC4-4	Normal	15.0	107,383,052	97.12%	38.8	85.2
OC10	Tumor	17.9	128,177,434	96.15%	38.4	78.9
OC12	Tumor	21.8	155,870,552	96.69%	38.5	76.5
OC14	Tumor	19.3	137,812,914	96.77%	38.6	73.6
OC16	Tumor	25.0	179,129,860	97.17%	38.8	84.6
OC18	Tumor	22.1	157,921,106	96.75%	38.6	83.2
OC2	Tumor	10.8	77,256,646	97.42%	39.0	86.9
OC20	Tumor	20.6	147,230,022	96.30%	38.5	83.1
OC22	Tumor	21.8	155,889,198	95.89%	37.9	81.5
OC2-2	Tumor	10.2	72,860,692	97.16%	38.9	85.6
OC24	Tumor	21.3	152,777,822	96.32%	38.1	79.5
OC26	Tumor	21.2	151,406,780	96.02%	38.0	81.4
OC28	Tumor	21.6	154,492,248	96.56%	38.2	84.3
OC30	Tumor	18.1	129,730,690	96.20%	38.0	78.2
OC31	Tumor	17.3	123,847,934	95.97%	37.8	80.5
OC33	Tumor	20.8	148,796,470	95.33%	37.5	82.4
OC35	Tumor	18.7	133,516,188	96.00%	37.7	82.3
OC37	Tumor	22.8	163,445,804	94.02%	36.9	83.2
OC39	Tumor	13.5	96,629,746	93.91%	36.9	82.3
OC41	Tumor	21.0	150,167,574	92.39%	36.5	78.6
OC43	Tumor	17.3	123,902,488	93.28%	36.8	78.5
OC47	Tumor	18.6	133,365,106	93.75%	36.9	78.1
OC49	Tumor	17.1	122,563,094	93.50%	36.9	76.8
OC51	Tumor	17.2	123,043,836	95.11%	37.3	87.1
OC53	Tumor	11.2	80,194,242	92.97%	36.6	73.7
OC55	Tumor	12.9	92,270,002	92.13%	36.4	78.4
OC6	Tumor	30.1	215,722,162	97.98%	38.7	82.9
OC8	Tumor	18.9	135,648,544	96.40%	38.4	75.6

Table S3 Sequences of primers and siRNAs used in the study

Oligo ids	Sequence(5'-3')
GAPDH F	AGCTCACTGGCATGGCCTTC
GADPH R	CGCCTGCTTCACCACCTTCT
β-actin F	GGACTTCGAGCAAGAGATGG
β-actin R	AGCACTGTGTTGGCGTACAG
circFBXO7 F	ATGCCGGAGAAGTGGAAGTT
circFBXO7 R	GCGCTGGAATGTCATCTTGA
Linear-FBXO7 F	CTGCGTGATTTTCGAGACAATACTG
Linear-FBXO7 R	TTGGGATAGAATGGAATGGTGTGA
Linear-FBXO7(exon) F	CCCAATCAGTTCACTCATTCCTGG
Linear-FBXO7(exon) R	CTGGGTCTAAAGGGAAATCTGTC
ATXN1 F	TCGGTGGAGCTTGGTTTACAA
ATXN1 R	GGGAGGACCCAATGAACTGG
ZEB1 F	GATGATGAATGCGAGTCAGATGC
ZEB1 R	ACAGCAGTGTCTTGTTGTTGT
FAM171A1 F	GCAGATGCGCTCATCGAGAT
FAM171A1 R	GCCCAGCTTATACTGGAACTTG
MTSS1 F	CAGTCCCAGCTTCGGACAAC
MTSS1 R	TGAGAGCAGATCCAATCTCCC
Negative control siRNA	UUCUCCGAACGUGUCACGUTT
CircFBXO7 siRNA1	UGAUUGUUGUAAAUGUUCUTT
CircFBXO7 siRNA2	AAAUGUUCUAAUACCCGAUTT
MTSS1 siRNA3	CCAGACUACGCUCAUUAUUTT
MTSS1 siRNA7	GGACUUGAAAGGUUCUGAUTT

Table S4 Quality control metrics of ployA-enriched RNA-seq libraries in this study

Sample ID	Yield (Gb)	# Reads	% of >= Q30 Bases	Mean Quality Score	mapping rate (%)
A2780-NC-1	9.0	61,690,386	95.8	37.5	95.0
A2780-NC-2	8.2	55,631,590	95.9	37.5	95.6
A2780-NC-3	11.0	75,404,538	96.4	37.7	97.1
A2780-OV-1	9.3	63,859,244	96.2	37.6	95.2
A2780-OV-2	9.5	64,507,248	96.2	37.6	95.8
A2780-OV-3	9.7	66,329,288	96.2	37.6	94.2
MADH2774-NC-1	9.4	64,504,280	96.7	37.8	94.9
MADH2774-NC-2	6.7	46,091,886	96.5	37.7	96.7
MADH2774-NC-3	10.0	68,689,436	96.0	37.5	96.7
MADH2774-OV-1	6.6	45,141,504	96.3	37.6	96.2
MADH2774-OV-2	8.5	58,837,950	96.3	37.7	96.6
MADH2774-OV-3	7.7	52,982,794	96.2	37.6	96.8

Table S5 Information of antibodies used in the study

Antibodies	Company	Catelogue#	Species
β -actin	BIOKER	BK7018	rabbit
GAPDH	Diagbio	db106	rabbit
MTSS1	abclonal	A11697	rabbit
VIM	CST	5741	rabbit
SNAIL1	CST	3879	rabbit
β -catenin	CST	8480s	rabbit
p-GSK3 β (phosphor S9)	Diagbio	db1715	rabbit

A

**ID**	hsa_circ_0001222
**Alias**	hsa_circ_001284
**Position**	chr22:32874967-32881196
**Strand**	+
**Genomic length**	6229
**Spliced sequence length**	665
**Annotation**	ANNOTATED<br>CDS<br>coding<br>INTERNAL<br>OVCODE<br>OVEXON
**Repeats**	None
**Best transcript**	NM_012179
**Gene symbol**	FBXO7

B

TTACATACCTCAGGGCACCGAAGCCAAAGCACTGTCCATGCCGGAGAAGTGGAAGTTGAGCGGGGTGTA
TAAGCTGCAGTACATGCATCCTCTCTGCGAGGGCAGCTCCGCTACTCTCACCTGTGTGCCTTTGGGAAAC
CTGATTGTTGTAAATGTTCTAATACCCGATTTACAATTACATTGAACTACAAGGATCCCCTCACTGGAGATG
AAGAGACCTTGGCTTCATATGGGATTGTTTCTGGGGACTTGATATGTTTGATTCTTCAAGATGACATTCCA
GCGCCTAATATACCTTCATCCACAGATTCAGAGCATTCTTCACTCCAGAATAATGAGCAACCCTCTTTGGC
CACCAGCTCCAATCAGACTAGCATGCAGGATGAACAACCAAGTGATTCATTCCAAGGACAGGCAGCCCA
GTCTGGTGTTTGGAATGACGACAGTATGTTAGGGCCTAGTCAAAATTTTGAAGCTGAGTCAATTCAAGAT
AATGCGCATATGGCAGAGGGCACAGGTTTCTATCCCTCAGAACCCATGCTCTGTAGTGAATCGGTGGAAG
GGCAAGTGCCACATTCATTAGAGACCTTGTATCAATCAGCTGACTGTTCTGATGCCAATGATGCCTTGATA
GTGTTGATACATCTTCTCATGTTGGAGTCAGG

Figure S1 Genomic information of circFBXO7. (**A**) circBase annotation of circFBXO7 (ID: hsa_circ_0001222). (**B**) Sequence of full-length circFBXO7 from Sanger sequencing.

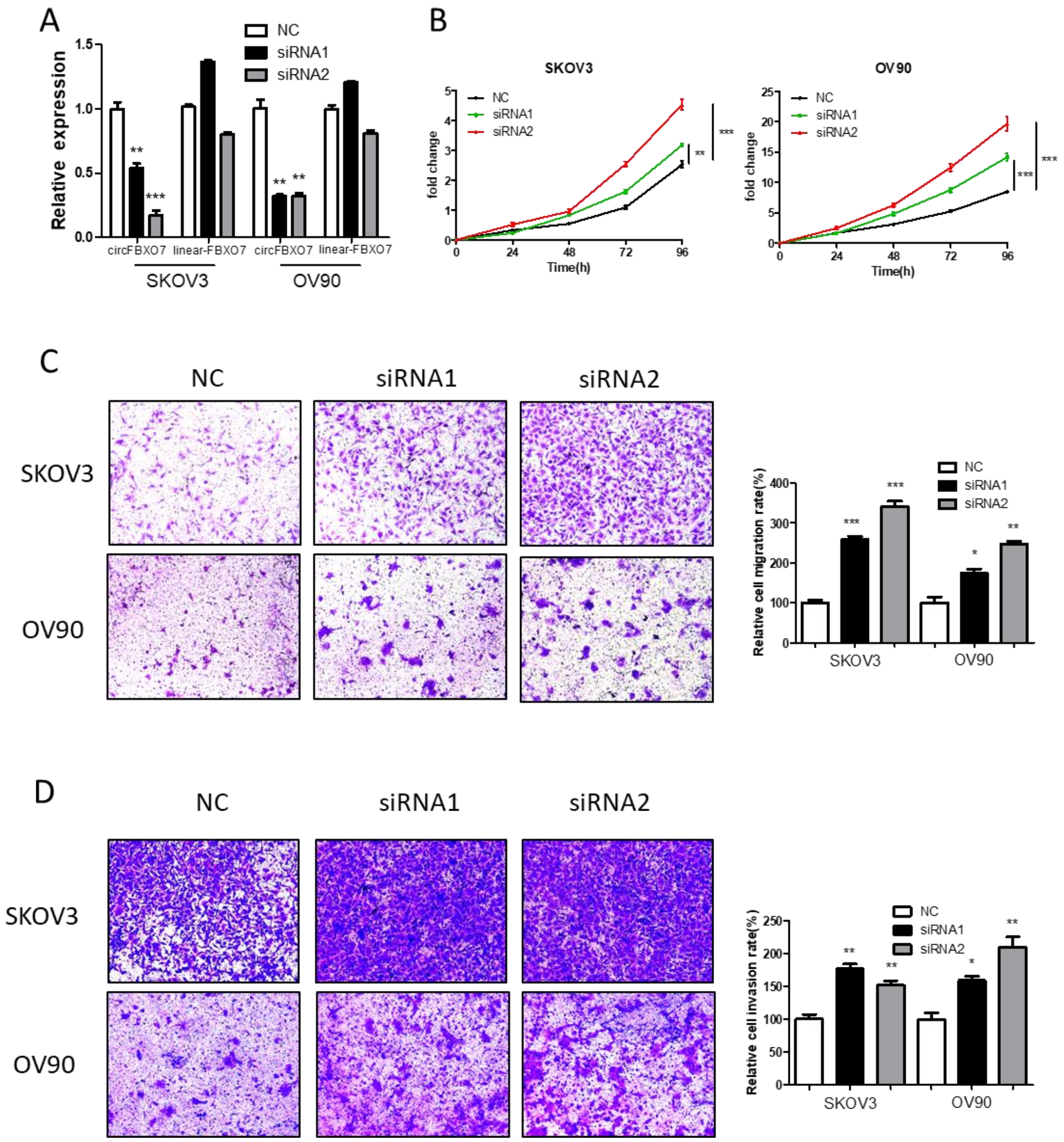

Figure S2 circFBXO7 exerts tumor suppressive effects in ovarian cancer cells. (**A**) qRT-PCR analysis of circFBXO7 and linear-FBXO7 expression in SKOV3 and OV90 cells transfected with circFBXO7 siRNA and negative control siRNA. (**B**) Growth curve of SKOV3 and OV90 cells transfected with circFBXO7 siRNA and negative control siRNA, as assessed by CCK-8 assays. (**C**) Migration ability of SKOV3 and OV90 cells transfected with circFBXO7 siRNA and negative control siRNAs, as assessed by transwell assays. (**D**) Invasion ability of SKOV3 and OV90 cells transfected with circFBXO7 siRNA and negative control siRNAs, as assessed by transwell assays.

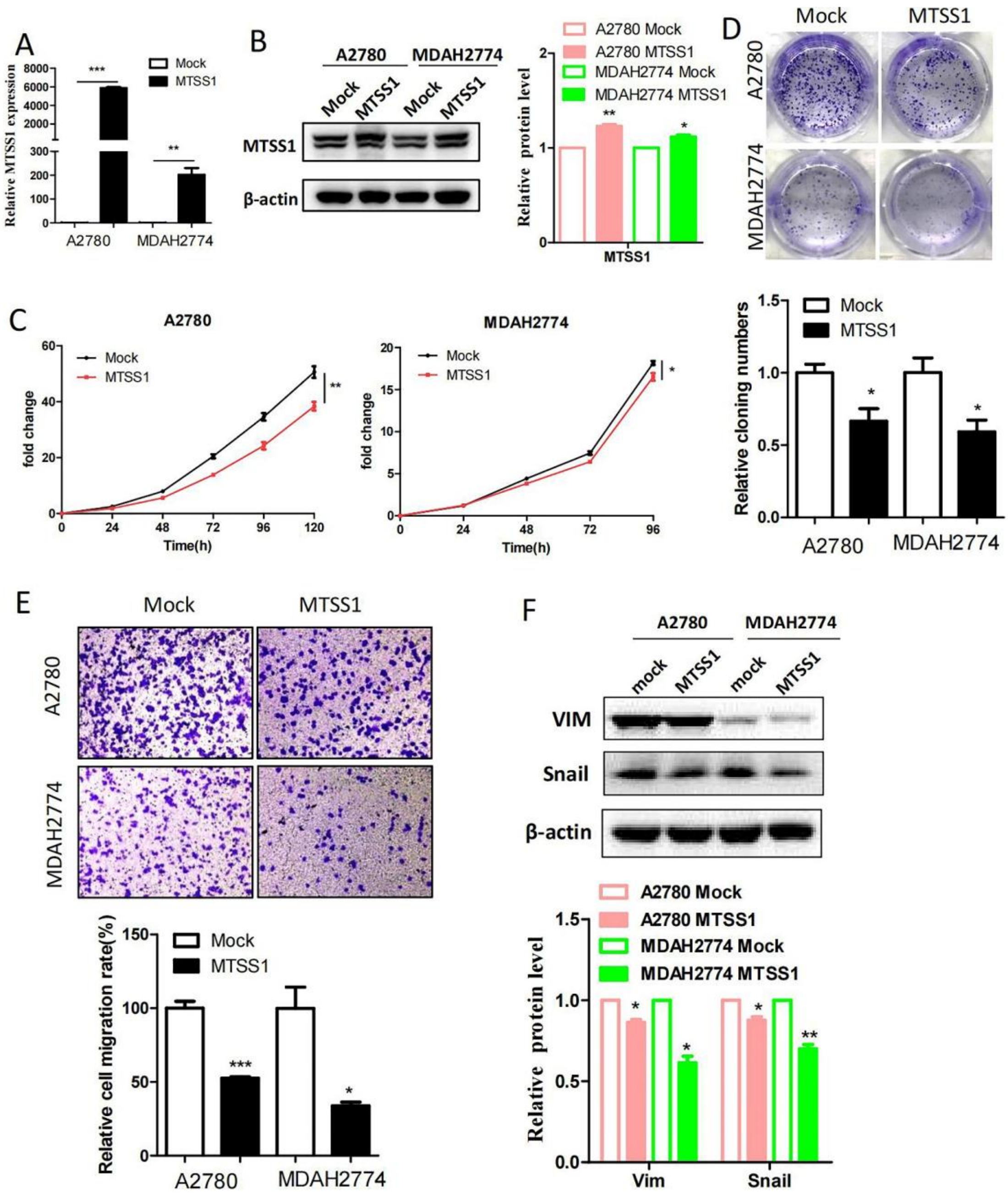

Figure S3 MTSS1 inhibits ovarian cancer cell proliferation and migration. (**A**) qRT-PCR analysis of MTSS1 expression in A2780 and MDAH2774 cells transfected with MTSS1 overexpression and mock plasmids. (**B**) Western blot analysis of the expression of MTSS1 in A2780 and MDAH2774 cells transfected with MTSS1 overexpression and mock plasmids. (**C**) Growth curve of A2780 and MDAH2774 transfected with MTSS1 overexpression and mock plasmids, as assessed by CCK-8 assays. (**D**) Proliferation of A2780 and MDAH2774 cells transfected with MTSS1 overexpression and mock plasmids, as determined by colony formation assay. (**E**) Migration ability of A2780 and MDAH2774 cells transfected with MTSS1 overexpression and mock plasmids. (**F**) Western blot analysis of the expression of VIM and snail in A2780 and MDAH2774 cells transfected with MTSS1 siRNA and negative control siRNA. Student's two-sided t tests were used. *** $P < 0.001$, ** $P < 0.01$, * $P < 0.05$.

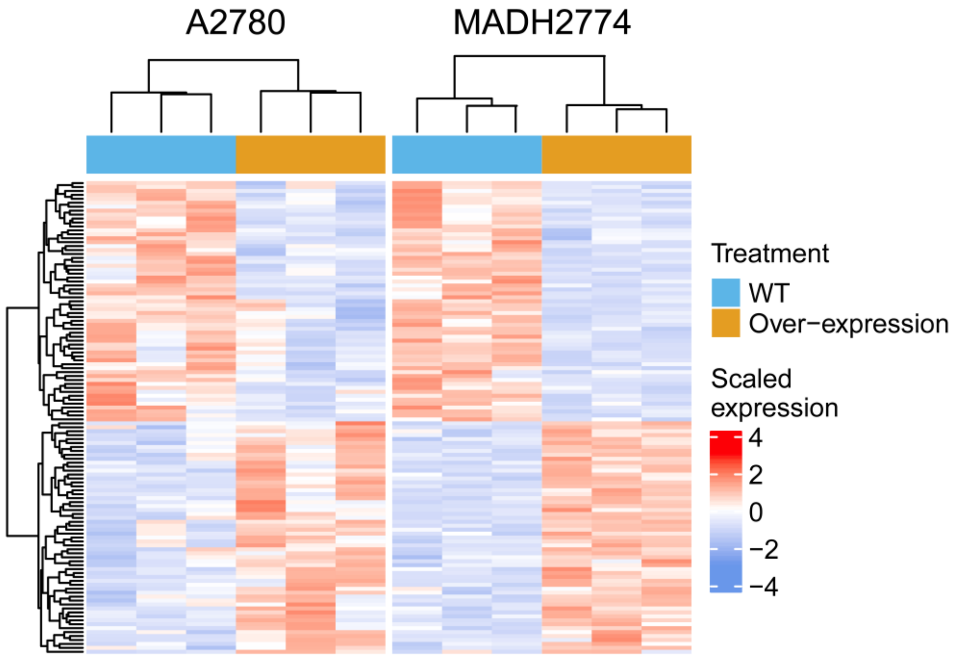

Figure S4 Heat map of differentially expressed genes between circFBXO7-overexpressing and control ovarian cancer cells.

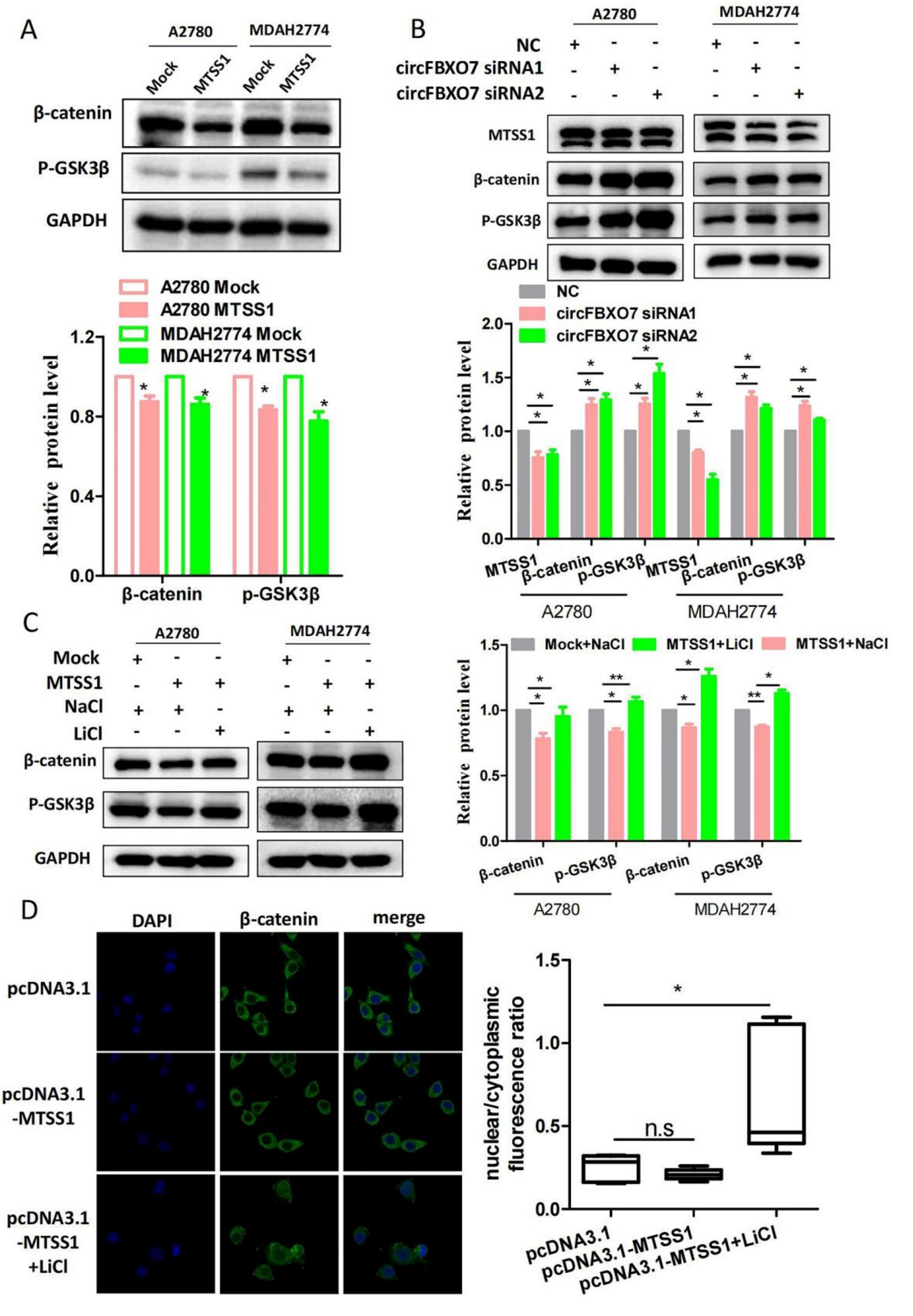

Figure S5 circFBXO7/MTSS1 is involved in the regulation of Wnt/β-catenin signaling. (**A**) Western blot analysis of the expression of wnt/β-catenin and p-GSK3β in A2780 and MDAH2774 cells transfected with MTSS1 overexpression and mock plasmids. (**B**) Western blot analysis of the expression of β-catenin and p-GSK3β in A2780 and MDAH2774 cells

transfected with circFBXO7 siRNA or negative controls. (**C**) Western blot analysis of the expression of β-catenin and p-GSK3β in MTSS1 overexpression A2780 and MDAH2774 cells transfected with LiCl, NaCl, or mock plasmids. (**D**) Representative images of IF micrographs of the subcellular localization and expression of β-catenin (green) in MTSS1 overexpression MDAH2774 cells transfected with LiCl. Nuclei were counterstained with DAPI (blue). Student's two-sided t tests were used. *** $P < 0.001$, ** $P < 0.01$, * $P < 0.05$.

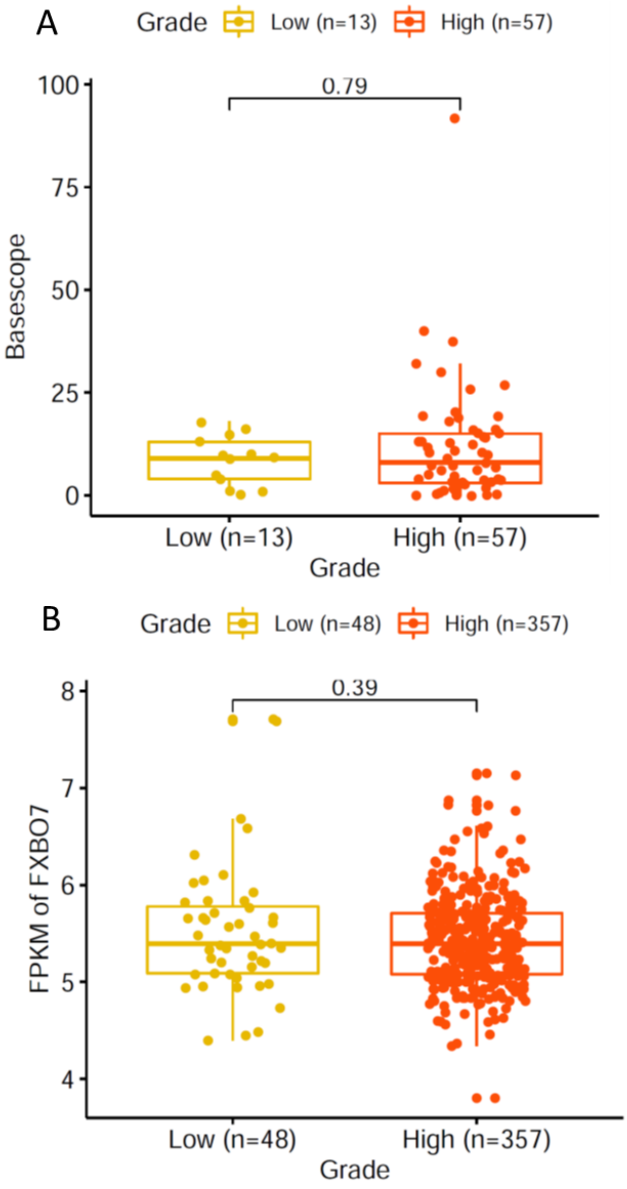

Figure S6 circFBXO7 and FBXO7 expression in low- and high-grade ovarian tumors. (**A**) CircFBXO7 expression in our FFPE cohort. (**B**) FBXO7 expression in the TCGA cohort.
